# Uncertainties of particulate organic carbon concentrations in the mesopelagic zone of the Atlantic ocean

**DOI:** 10.12688/openreseurope.13395.2

**Published:** 2021-09-06

**Authors:** Paul Strubinger Sandoval, Giorgio Dall'Olmo, Keith Haines, Rafael Rasse, Jelizaveta Ross

**Affiliations:** 1 Plymouth Marine Laboratory, Prospect Place, Plymouth, PL1 3DH, UK; 2 National Centre for Earth Observation, Plymouth, PL1 3DH, UK; 3 University of Reading, Reading, Berkshire, RG6 6AH, UK; 4 National Centre for Earth Observation, Reading, RG6 6AH, UK; 5 Sorbonne Universités, Laboratoire d’Océanographie de Villefranche, Institut de la Mer de Villefranche, Villefranche-sur-Mer, 06230, France

**Keywords:** particulate organic carbon, POC, uncertainty, uncertainty budget, mesopelagic, Atlantic Meridional Transect

## Abstract

Measurements of particulate organic carbon (POC) in the open ocean provide grounds for estimating oceanic carbon budgets and for modelling carbon cycling. The majority of the published POC measurements have been collected at the sea surface. Thus, POC stocks in the upper layer of the water column are relatively well constrained. However, our understanding of the POC distribution and its dynamics in deeper areas is modest due to insufficient in POC measurements. Moreover, the accuracy of published POC estimates is not always quantified, and neither is it fully understood. In this study, we determined the POC concentrations of samples collected in the upper 500 m during an Atlantic Meridional Transect and described a method for quantifying its experimental uncertainties using duplicate measurements. The analysis revealed that the medians of the total experimental uncertainties associated with our POC concentrations in the productive and mesopelagic zones were 2.5(±1.2) mg/m ^3^ and 2.6(±0.6) mg/m ^3^, respectively. In relative terms, these uncertainties corresponded to ~14% and ~ 35% of POC concentrations, respectively. However, despite our best efforts, we could explain only ~ 21% of the total experimental POC uncertainty. The potential sources of this unexplained portion of uncertainty are discussed.

## 1. Introduction

Particulate organic carbon (POC) is operationally defined as the non-carbonate and combustible carbon matter in particulate form that is retained by a filter of a typical nominal pore size of 0.7 *µ*m ( Kharbush *et al.,* 2020, IOCCG POC Protocols). In the open-ocean, pelagic POC mainly includes suspended detrital matter (i.e., remains of dead organisms) and living organisms (i.e. phytoplankton, heterotrophic bacteria and zooplankton) ( Gardner *et al.,* 2006; Turnewitsch *et al.,* 2007).

POC constitutes the third most abundant carbon pool ( *∼*2 Pg C, Brewin *et al.,* 2021; Stramska & Cieszyńska, 2015) in the ocean, the others being the dissolved inorganic ( *∼*38,000 Pg C, Hedges, 1992) and dissolved organic ( *∼*662 Pg C, Hansell & Carlson, 2013) and the particulate inorganic carbon pools ( *∼*0.03 Pg C, Hopkins *et al.,* 2019). POC has one of the highest turnover rates among the pools of carbon in the ocean ( Brewin *et al.,* 2021; Sarmiento & Gruber, 2006) and the efficiency with which it is transferred from the upper to the deep ocean and sediments contributes to controlling atmospheric CO _2_ concentrations ( Kwon *et al.,* 2009; Parekh *et al.,* 2006).

In the last decades, several studies have collected relatively extensive measurements of POC, leading to progress in understanding the nature of POC and the processes controlling its spatio-temporal distribution ( Aumont *et al.,* 2017; Bishop & Wood, 2008; Bishop & Wood, 2009; Cetinić *et al.,* 2012; Gardner *et al.,* 2003; Gardner *et al.,* 2006; Henson *et al.,* 2012; Kharbush *et al.,* 2020; Kiko *et al.,* 2017; Lam *et al.*, 2011; Moran *et al.,* 1999; Rasse *et al.,* 2017; Turnewitsch *et al.,* 2007; Wangersky, 1976). However, even though much effort has been invested in developing methods for determining POC ( Gardner *et al.,* 2000; Gardner *et al.,* 2003; Gardner *et al.,* 2006; Liu *et al.,* 2005; Liu *et al.,* 2009; Moran *et al.,* 1999; Wangersky, 1974; Wangersky, 1976), we still have a relatively rudimentary understanding of the uncertainties associated with the different steps of the analysis and, therefore, with the resulting POC estimates. This is a problem because identifying and quantifying the uncertainties of POC will ultimately allow the community to further understand how and why POC varies in the ocean. In addition, by better characterising POC uncertainties and the major sources of these uncertainties, we might improve the method for determining POC.

The objectives of this study were 1) to present a method to experimentally quantify the uncertainties of POC measurements based on duplicate filters in both pelagic and deep ocean layers; 2) to model POC uncertainty based on assumed sources of uncertainty affecting the determination of POC; and 3) to compare modelled and experimental uncertainties. Our results show that modelled uncertainties accounted for only a small fraction of the experimental POC uncertainties, suggesting that sources of uncertainty different from those considered in our analysis controlled the uncertainty of our POC determinations. Identifying, characterising and minimising these additional uncertainties will lead to improved measurements of POC and a better understanding of its variability and the role it plays in the ocean carbon cycle.

## 2. Data and methods

At the time when the data presented in this study were collected, the best known and accepted protocol for determining POC was the one established in the mid 90’s for The Joint Global Ocean Flux Study (JGOFS) ( Knap *et al.,* 1996). Studies that appeared after the JGOFS protocol identified additional potentially important sources of uncertainty in the POC determination, as for example the adsorption of DOC onto the POC filters or the volume of water needed to minimise uncertainties when POC values are low ( Cetinić *et al.,* 2012; Gardner *et al.,* 2003; Gardner *et al.,* 2006; Moran *et al.,* 1999; Stramski *et al.,* 2008). In an attempt to quantify and minimize the uncertainties of the POC concentrations determined in the present study, we modified the sampling, processing and analysis described in the JGOFS protocol. Modifications, described in more detail in Section 2.1, Section 2.2 and Section 2.3, included the sampling of different volumes of water in accordance to expected in-situ concentrations, and using different types of blanks to quantify the dissolved organic carbon and any contamination due to the acidification step. Yet, minimising all uncertainties proved difficult. More recently, a NASA-led team has been developing a revised and considerably more detailed POC protocol (currently in draft version) to support the validation of ongoing and upcoming ocean-colour satellite missions (see Chaves *et al.*, 2021).

### 2.1 Sampling

**Figure 1.**
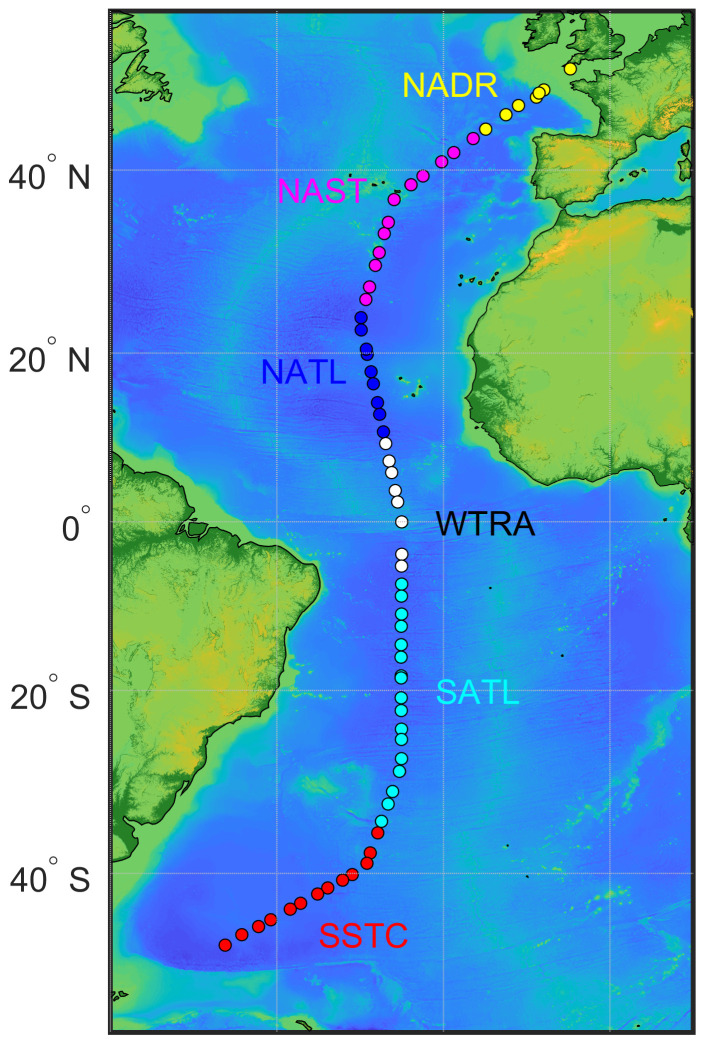
Track of AMT-24 cruise with the locations of 67 stations where samples for particulate organic carbon concentration were collected. Colour coding of the stations represents biogeographical provinces that were sampled: the North Atlantic Drift Province (NADR), the North Atlantic Subtropical Gyral Province (NAST), the North Atlantic Tropical Gyral Province (NATL), the Western Tropical Atlantic Province (WTRA), the South Atlantic Gyral Province (SATL), and the South Subtropical Convergence Province (SSTC).

Water samples were collected at 67 stations during the 24 *^th^* Atlantic Meridional Transect (AMT-24) aboard the RRS James Clark Ross from September 25 *^th^* to November 1 *^st^*, 2014 (see Figure 1). Two casts were completed every day (weather permitting): one pre-dawn and the other around solar noon. Different ecological provinces ( Longhurst, 2007) were sampled: the North Atlantic Drift Province (NADR), the North Atlantic Subtropical Gyral Province (NAST), the North Atlantic Tropical Gyral Province (NATL), the Western Tropical Atlantic Province (WTRA), the South Atlantic Gyral Province (SATL), and the South Subtropical Convergence Province (SSTC).

At each station, water samples were collected from six depths in the upper 500 m using 20-l Niskin bottles (Ocean Test Equipment Inc., Standard ES, Model 115) that were installed on a bespoke stainless-steel frame. The sampling procedure did not account for dregs, the rare large particles that might have been under sampled by Niskin bottles ( Gardner *et al.,* 2006). Water samples were transferred from the Niskin bottles into six 15-l HDPE carboys, which were pre-washed with 10% HCl and then with sample water prior to use. The carboys were taken to the on-board laboratory, where water samples were transferred into six *∼*2-l narrow-mouth amber bottles (Thermo Scientific Nalgene) and filtered through pre-combusted (450°C for five hours) 25 mm Whatman glass fiber filters (GF/F, nominal pore size 0.7 *µ*m) at low vacuum ( *∼*125 mm Hg) by inverting the bottles into a standard funnel setup. Each of the bottles was also pre-washed with sample water prior to use. To keep the water samples homogenised, carboys were gently shaken before pouring into the bottles. The volume of sample water filtered for each POC measurement varied between *∼*1 and *∼*8 liters, and it was adjusted according to the expected concentration of POC in the respective environment. Thus, each 2-l bottle was typically re-filled (up to four times) during filtration to achieve the total volume for any given sample. No measures were taken to prevent atmospheric contamination during filtration.

We implemented the "double-filter" technique advocated by Banoub & Williams (1972); Feely (1974); Kinney *et al.* (1971); Loder & Hood (1972); Moran *et al.* (1999); Smith *et al.* (1996). These authors suggested using two stacked filters for each estimate of POC concentration. The upper filter was used to collect particles and adsorbed dissolved organic carbon (uncorrected POC sample, henceforth referred to as "uPOC") while the lower filter quantified the dissolved organic carbon adsorbed onto GF/F filters (adsorbed DOC blank, henceforth referred to as "aDOC") (see Table 1 for a list of variable names and their subscripts). After each filtration, uPOC and aDOC filters were removed from the filtration rig, wrapped into separate pre-combusted (450°C for five hours) aluminium foil envelopes, flash-frozen in liquid nitrogen, and then stored in a freezer at *−*80°C for post-cruise analysis.

**Table 1.** List of variables names and their subscripts.

Subscript	Variable Name	Units
POC	Particulate Organic Carbon	N/A
uPOC	Uncorrected Particulate Organic Carbon	N/A
aDOC	Adsorbed Dissolved Organic Carbon	N/A
*M*_uPOC_	Mass of organic carbon on uPOC	*µ*g
*M*_aDOC_	Mass of organic carbon on aDOC	*µ*g
*M*_cap_	Mass of organic carbon on tin capsules	*µ*g
*M*_ac_	Mass of organic carbon on acidified filters	*µ*g
*M*_nac_	Mass of organic carbon on non-acidified filters	*µ*g
$M_{\mathrm{uPOC}}^{\text{*}}$	Blank-corrected mass of organic carbon on uPOC	*µ*g
$M_{\mathrm{aDOC}}^{\text{*}}$	Blank-corrected mass of organic carbon on aDOC	*µ*g
*M*	Mass of particulate organic carbon	*µ*g
*C*	Concentration of particulate organic carbon	mg/m ^3^
*D*_1_	Carbon concentration of the first duplicate	mg/m ^3^
*D*_2_	Carbon concentration of the second duplicate	mg/m ^3^
$\bar{D}$	Average of POC concentration in the two duplicates	mg/m ^3^
∆	Scaled arithmetic difference between duplicate pairs	mg/m ^3^
∆ *_r_*	Scaled relative difference between duplicate pairs	dimensionless
*σ*_∆_	Uncertainty of each duplicate difference	mg/m ^3^
*σ _dr_*	Relative experimental uncertainties of duplicate values	dimensionless
*σ _∆r_*	Robust standard deviation of the relative duplicates pairs	dimensionless
*σ _r_*	Median of *σ _dr_*	dimensionless
*σ _C_*	Total experimental uncertainty of POC estimates	mg/m ^3^
*σ _V_*	Combined uncertainty of volume V	L
*σ*_*C*_( *V* )	Total uncertainty explained by uncertainty in volume	mg/m ^3^
*σ _M_*	Uncertainty of carbon mass predicted by the calibration equation (Prediction interval)	*µ*g
*σ*_*M*_P__	Uncertainty of carbon mass predicted by the calibration equation (standard law of propagation)	*µ*g
*σ*_*C*_( *M*)	Total uncertainty explained by uncertainty in regression models	mg/m ^3^
*σ*_*C*_( *η*)	Total uncertainty explained by uncertainty in handling	mg/m ^3^
*L _C_*	Critical value	*µ*g
*L _D_*	Detection limit	*µ*g

At each cast, a duplicate sample from a randomly chosen depth was collected to assess the uncertainty of our method. Sample water for each pair of duplicate measurements was taken from the same Niskin bottle. In total, we collected 392 uPOC samples with their corresponding aDOC blanks and 57 duplicate uPOC samples with their related aDOC blanks. In theory, a larger number of replicates per sample will provide more robust estimates of the POC uncertainty. Yet, in our case duplicates were chosen as a compromise between statistical robustness, the water available from the rosette, and the time required to collect and analyse the samples.

### 2.2 Laboratory sample handling

All the filters were processed in 16 separate batches or CHN runs. Each run consisted of (1) uPOC filters and their corresponding aDOC blanks from multiple casts, (2) duplicate uPOC and aDOC filters from these casts used to estimate total experimental uncertainties, (3) empty tin capsules, acidified and non-acidified filter blanks (detailed below) used to estimate uncertainties related to the sample handling in the lab and systematic biases, and (4) standards used to calibrate the CHN analyser (see Section 2.3 for details).

Acidification at room temperature for a period of 12 to 16 hours was used to remove the inorganic carbon accumulated on the uPOC and aDOC filters. To do this, a crucible containing a small amount of 37% HCl (Sigma Aldrich, High Purity, 08256-500ml F) was located in the middle of a glass desiccator. The uPOC filters and aDOC blanks from each batch were removed from their aluminium envelopes, placed into individual acid-washed glass vials, and positioned around the crucible in the desiccators. In contrast to adding an aliquot of a dilute acid solution directly onto the filters, the technique of exposing them to acid fumes is expected to homogenize the effect of the acid on all the filters within a desiccator and to avoid losses of organic particles ( Martin *et al.,* 1993). To minimise differences in contamination between corresponding uPOC and aDOC samples, paired uPOC and aDOC filters were acidified in the same desiccator. Duplicate filters were acidified in different desiccators.

To account for any potential contamination during acid fuming, we introduced an additional type of pre-combusted (450°C for five hours) 25 mm Whatman GF/F filter blank. Three of these filter blanks were placed into each desiccator, then acidified and processed along with uPOC filters and aDOC blanks, while three other filter blanks were kept clean and dry outside of the desiccators.

After the acidification phase, all the filters, including the acidified uPOC filters, aDOC blanks, and acidified and non-acidified filter blanks were dried in an oven at 60°C for several hours. Subsequently, the acidified and non-acidified filters and blanks were wrapped into individual tin capsules (Pressed, Standard Clean, 10 *×* 10 by OEA Labs) and analysed for carbon. An increment on the acidified filter blank masses in comparison with the non-acidified ones would indicate contamination during sample acidification and drying.

### 2.3 Determination of POC

The mass of carbon contained on filters was determined by high-temperature combustion ( Gordon & Sutcliffe, 1974; Menzel & Vaccaro, 1964; Sharp, 1974; Wheeler *et al.,* 1997) using a CHN analyser (FlashEA *^TM^* 1112 Elemental Analyzer, with helium CP grade N5.0 as carrier gas). Filters were processed in accordance with the manufacturer’s manual ( ThermoQuest, 1999). In every CHN run, a new reaction tube was used. The extracted CO _2_ was measured by a thermal conductivity detector.

As samples are analysed, the reaction tube of FlashEA *^TM^* CHN analyser gradually fills up with the combusted tin capsules and filters. Thus, as the CHN run proceeds and the reaction tube fills up, a variation in the instrument combustion efficiency can occur with time. To stabilise the combustion efficiency of the instrument throughout each run, we performed constant adjustments to the “sample delay” parameter, which represents the time that it takes the CHN analyser to combust each sample, to deliver CO _2_ released from the sample to the detector, and to run the analysis.

The CHN analyser was calibrated during each run using two sets of pre-weighted (Sartorius MC5 high-accuracy microbalance, calibrated yearly) acetanilide standards (C = 71.09%, N = 10.36%, OEA Labs, R66005) contained in tin capsules. The first set of 11 standards covered the entire range of expected masses of carbon on our uPOC and aDOC filters (5 – 300 *µ*g) and it was analysed immediately prior to processing the sample filters. We will refer to this set of standards as the *calibration standards*. The second set of standards was processed alongside the filters (one standard after every six filters) to validate the initial calibration throughout filter processing. We will refer to the second set of standards as the *stability standards*.

In the absence of any instrumental drift, we expected the calibration coefficients derived from both sets of standards from the same CHN run to be statistically indistinguishable. However, during seven out of 16 runs the CHN analyser was unstable for unknown reasons and the calibration coefficients derived from the two sets of standards differed significantly. Thus, we decided to use for all CHN runs, both types of standards, *calibration* and *stability*, to develop the relationship between the response of the CHN analyser and the mass of carbon on processed filters.

The mass of carbon *M* on the *i ^th^* filter, processed during the *k ^th^* CHN run, was estimated by a linear regression model using a robust fitting method:


$$M_{ik}=m_{k}x_{ik}+b_{k},\;(1)$$

where *x* represents the output signal from the CHN analyser, *m* and *b* represent the slope and the intercept of the regression line, respectively. The intercept was removed from the model when it was not statistically significant (p- value > 0.05).

For each CHN run, we estimated the mass of organic carbon contained on uPOC ( *M*_uPOC_), aDOC ( *M*_aDOC_) filters, tin capsules ( *M*_cap_), and acidified ( *M*_ac_) and non-acidified ( *M*_nac_) GF/F filters using Equation 1. These *M*_uPOC_ and *M*_aDOC_ values, however, do not represent the true load of particulate organic carbon contained in the corresponding water samples as these values may be affected by biases, i.e., contamination during the acidification step, residual organic carbon on the tin capsules, and on the combusted GF/F filters). Therefore, the blank-corrected mass of organic carbon from the *i ^th^* uPOC and aDOC filters, which were acidified together in the *j ^th^* desiccator and processed in the *k ^th^* CHN run ( $M_{\text{uPO}{\text{C}}_{ijk}}$ and $M_{\text{aDO}{\text{C}}_{ijk}}$ , respectively), must be written as (notation as in Turnewitsch *et al*., 2007):


$$M_{\text{uPO}{\text{C}}_{ijk}}^{*}=M_{\text{uPO}{\text{C}}_{ijk}}–{\bar{M}}_{\text{ca}{\text{p}}_{k}}–({\bar{M}}_{\text{a}{\text{c}}_{jk}}–{\bar{M}}_{\text{na}{\text{c}}_{k}})$$

$$M_{\text{aDO}{\text{C}}_{ijk}}^{*}=M_{\text{aDO}{\text{C}}_{ijk}}–{\bar{M}}_{\text{ca}{\text{p}}_{k}}–({\bar{M}}_{\text{a}{\text{c}}_{jk}}–{\bar{M}}_{\text{na}{\text{c}}_{k}}),$$

where ${\bar{M}}_{\text{a}{\text{c}}_{jk}}$ is the average carbon mass of the three filter blanks acidified in the same desiccator *j ^th^* as the *i ^th^* filter and ${\bar{M}}_{\text{na}{\text{c}}_{k}}$ and ${\bar{M}}_{\text{ca}{\text{p}}_{k}}$ are the average carbon mass of the three non-acidified filter blanks and the average carbon mass of three tin capsules, respectively.

For each pair of uPOC and aDOC filters, the mass of POC, *M*, was determined as


$$M_{ijk}=M_{\text{uPO}{\text{C}}_{ijk}}^{*}-M_{\text{aDO}{\text{C}}_{ijk}}^{*}.\;(2)$$

We assumed that uPOC and aDOC filters had adsorbed the same amount of DOC, and their contamination due to sample handling during the CHN analysis was equal to the average mass of the three acidified filter blanks. Hence, the subtraction in Equation 2 removed various systematic biases from the final estimates of the mass of POC. Note that because we processed corresponding pairs of uPOC and aDOC filters in the same desiccator and during the same CHN run, Equation 2 is equivalent to:

$$M_{ijk}=M_{\text{uPO}{\text{C}}_{ijk}}-M_{\text{aDO}{\text{C}}_{ijk}}.\;(3)$$

To determine the POC concentration, *C*, for each water sample, we divided *M* by the volume of water, V, filtered for each sample:

$$C_{ijk}=\frac{M_{ijk}}{V}.\;(4)$$

### 2.4 Uncertainty analysis

The standard law of propagation of uncertainty ( JCGM, 2008) was used throughout our uncertainty calculations and we recall it here for the reader:

$$\sigma_{y}^{\text{2}}=\sum_{i=1}^{N}{\left(\frac{\partial y}{\partial x_{i}}\right)}^{2}\sigma_{x_{i}}^{\text{2}}+2\sum_{i=1}^{N-1}\sum_{j=i+1}^{N}\;\frac{\partial y}{\partial x_{i}}\frac{\partial y}{\partial x_{j}}\sigma_{x_{i}}\sigma_{x_{j}}r_{\left(x_{i},x_{j}\right)},\;(5)$$

where $\sigma_{y}^{2}$ is the total (or "combined") variance of the estimate *y* (POC, in our case), which is determined from the input quantities *x*_1_, *x*_2_, ..., *x _N_* through the functional relationship *y* = *f* ( *x*_1_, *x*_2_, ..., *x _N_*). The uncertainty of each of the input variables is denoted as $\sigma_{x_{i}}$ and their inter-dependencies are represented by the correlation coefficients *r*_( *x _i_*, *x _j_*)_. The total uncertainty is the positive square root of $\sigma_{y}^{2}$ .

***2.4.1 Total experimental uncertainty.*** We first estimated the total *experimental* uncertainties associated with our POC concentrations by analysing the measurements from the duplicate samples. These total experimental uncertainties are expected to represent the uncertainties arising from all (or at least most of) the steps required to estimate POC concentrations (i.e., from sample collection in the field to sample analysis in the laboratory). Below we show that these uncertainties depend on the POC concentration and therefore we present them as relative uncertainties. The median values of the relative uncertainties estimated from all duplicates will then be used to estimate the uncertainty of any sample collected during the cruise. Thus, this last step uses the statistics from all duplicates to estimate *population* uncertainties, rather than *per-sample* uncertainties.

Experimental uncertainties were estimated from the scaled arithmetic differences between the POC concentrations of the duplicates, *D*_1_ and *D*_2_:

$$\;\text{Δ}=\frac{D_{1}-D_{2}}{\sqrt{2}}.\;(6)$$

Since both *D*_1_ and *D*_2_ are uncertain and their uncertainties add in quadrature, to estimate the uncertainty in only one measurement, the difference of the duplicates was divided by $\sqrt{2}$ ( Hyslop & White, 2009).

**Figure 2.**
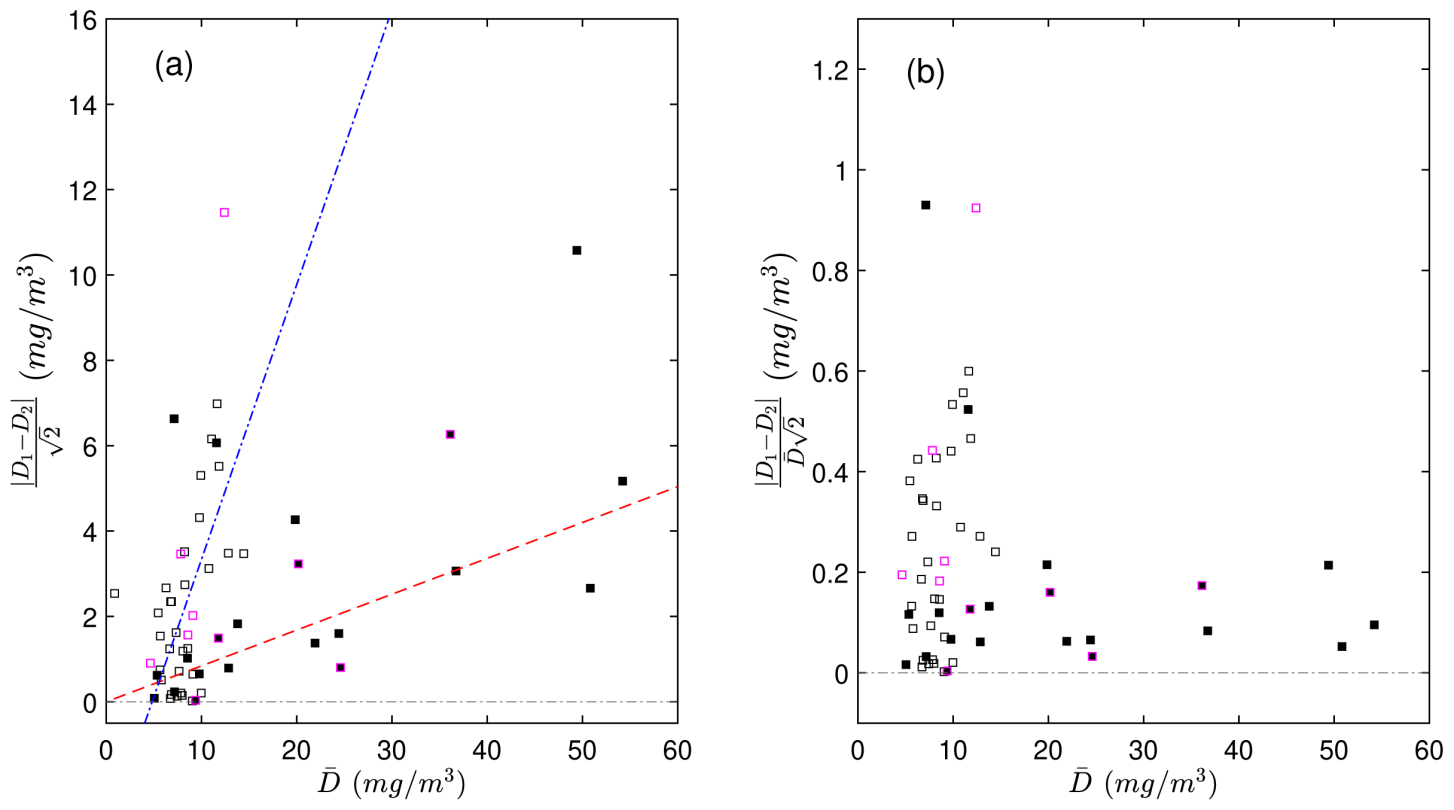
Scaled differences in duplicate particulate organic carbon (POC) measurements ( **a**) as a function of the mean value of each pair of duplicates ( $\bar{D}$ ). Scaled relative differences in duplicate POC measurements relative to $\bar{D}$ ( **b**). The absolute values were used to more easily demonstrate the dependency of the duplicate differences on POC. Black and white points represent duplicate measurements from the productive and mesopelagic zones, respectively. Points with magenta borders represent samples processed during CHN runs with highly uncertain calibrations. The red dashed and blue dash-dotted lines are the linear fits to the data in the productive and mesopelagic zones, respectively.

We found that the duplicate differences ∆ were positively related to POC concentration and that this dependency was different between the productive and mesopelagic zones ( Figure 2a). The correlation coefficient between ∆ and the average POC concentration of duplicates was 0.4 in the productive zone and 0.54 in the mesopelagic zone. To remove this dependency on concentration, we expressed the differences in duplicate measurements as a relative difference (∆ *_r_* = ∆/ $\bar{D}$ ), where $\bar{D}$ is the average of POC concentration in the two duplicates. Figure 2b confirms that, once normalised, the relative duplicate differences in the productive and mesopelagic zones did not depend on POC anymore. These findings imply that estimates of POC concentration from a specific zone should have the same relative uncertainty, *σ _r_*.

The total experimental uncertainty was computed by analysing the ∆ values as follows. The uncertainty of each duplicate difference, *σ*∆, depends on the unknown uncertainties of the individual duplicate estimates, $\sigma_{d_{1}}$ and $\sigma_{d_{2}}$ . By applying the law of propagation of uncertainty ( Equation 5) to Equation 6 and by assuming no correlation between the duplicate estimates, we find:

$$\sigma_{\text{Δ}}^{2}=\frac{\sigma_{d_{1}}^{2}+\sigma_{d_{2}}^{2}}{2}=\frac{{\left(\sigma_{d_{r}}D_{1}\right)}^{2}+{\left(\sigma_{d_{r}}D_{2}\right)}^{2}}{2}=\frac{\sigma_{d_{r}}^{2}(D_{1}^{2}+D_{2}^{2})}{2},\;(7)$$

where $\sigma_{d_{r}}$ is the relative uncertainty of the duplicate and we assumed that, as for the duplicate differences, also $\sigma_{d_{i}}$ (with *i* = 1, 2) needs to be expressed in relative terms (i.e., $\sigma_{d_{i}}$ = $\sigma_{d_{r}}$ *D _i_*).

By rearranging Equation 7, $\sigma_{d_{r}}$ can be expressed in terms of the uncertainty of the difference, *σ*_∆_:

$$\;\sigma_{d_{r}}=\frac{\sigma_{\text{Δ}}\sqrt{2}}{\sqrt{D_{1}^{2}+D_{2}^{2}}}\;(8)$$

Expressing *σ*_∆_ in relative terms yields:

$$\;\sigma_{d_{r}}=\frac{\sigma_{{\text{Δ}}_{r}}\bar{D}\sqrt{2}}{\sqrt{D_{1}^{2}+D_{2}^{2}}}.\;(9)$$

$\sigma_{\Delta_{r}}$ was then estimated as the robust standard deviation of the relative duplicates in each zone:

$$\;\sigma_{{\text{Δ}}_{r}}=\frac{{\text{P}}_{\text{84}}({\text{Δ}}_{r})-{\text{P}}_{\text{16}}({\text{Δ}}_{r})}{2},\;(10)$$

where P _84_ and P _16_ are the 84 ^th^ and 16 ^th^ percentiles of ∆ *_r_* ( Hyslop & White, 2009).

Finally, the total experimental uncertainty of POC concentration was estimated as:

$$\;\sigma_{C}\approx \sigma_{r}C,\;(11)$$

where *σ _r_* is the median of $\sigma_{d_{r}}$ , representing 14% and 35% uncertainties in POC concentrations for the productive and the mesopelagic zone, respectively.

### 2.5 Modeled uncertainty and uncertainty budget

In this section, we used a second method to model an independent estimate of the total uncertainty in POC concentrations. Specifically, the standard law of propagation of uncertainty was used to propagate the uncertainties associated with different steps of the POC determination. This independent model estimate of the POC uncertainty was then compared to the total experimental uncertainty derived from the duplicate measurements to assess the extent to which this latter theoretical calculation could reproduce the experimental uncertainties: the closer these estimates are, the more confident we can be in how we understand the measurement process and its uncertainties.

Equation 5 also allows one to estimate an uncertainty budget, that can be used to partition the total uncertainty into different contributions. Here, the relative contribution of each uncertainty source was computed as the ratio of the variance of each specific modeled uncertainty source divided by the total experimental variance (i.e., uncertainty squared). The sum of the modeled relative variances is an estimate of how well the modeled and total experimental uncertainties agree. The different relative uncertainty contributions can be ranked to identify the most uncertain steps in the methodology and prioritise improvements in the method. Table 2 presents the list of the sources of uncertainty that we were able to quantify.

**Table 2.** Sources of uncertainty that contribute to the total uncertainty of particulate organic carbon (POC) estimates and that we could quantify. More detailed explanation of calculations are given in Section 2.5. The median values of each uncertainty is given for the productive zone (PZ) and for the mesopelagic zone (MZ).

No	Source	Method of calculations	Values		Units
			PZ	MZ	
1	Volume	Volume uncertainty of each sample is equal to half graduation mark of the measuring cylinder used to estimate the volume of each bottle, multiplied by the number of bottles used during a given filtration	0.01	0.02	L
2	Calibration	Prediction intervals (PI) of the calibration curves of each CHN run	2.14	2.15	*µ*g
3	Sample handling during CHN analysis	Quantify the uncertainty that could arise from acidification and processing of sample and blank filters.	0.37	0.34	*µ*g
4	Total	Combined uncertainty calculated from POC duplicates	2.29	2.60	mg/m ^3^

***2.5.1 Uncertainty in volume.*** Errors in measuring the volume of sample seawater translate into uncertainties in POC concentration. Since each POC sample required from one to five bottles of sample seawater, each with a volume *V _n_*, the combined uncertainty of the total volume, *V*, of seawater used for a sample depended on the number of bottles, *n*, used and the uncertainty in volumetric measurements, *σ _V _n__*, of each bottle. *σ _V _n__* was set equal to half of a graduation mark of the measuring cylinder (uncertainty of 10 ml). The volume of each bottle (~2.2 l) was measured with a measuring cylinder multiple times.

Thus, the combined uncertainty of volume *V* can be expressed as

$$\;\sigma_{V}=\sqrt{\sum \sigma_{V_{n}}^{2}}.\;(12)$$

We estimated the uncertainty in POC concentration that could be explained by the uncertainty in volume, *σ _C_* ( *V*), by applying the propagation of uncertainty to Equation 4 and obtained:

$$\;\sigma_{C}(V)=\frac{M\sigma_{V}}{V^{2}}.\;(13)$$

***2.5.2 Uncertainty in mass predicted by the calibration equation***. The uncertainty of carbon mass *M _ik_* predicted using the calibration equation ( Equation 1) was expected to be one of the largest contributors to the uncertainty budget because it determined the quality and reliability of the prediction of the masses of carbon in all components of the analysis. To estimate the uncertainties of the carbon masses predicted by our calibration equations, *σ _M_*, we used 68% prediction intervals (PIs) as they serve in a normal distribution as one standard deviation. A PI is defined similarly to the better known confidence interval. However, the PI is more appropriate for quantifying the uncertainty of the calibration process because it represents the expected uncertainty of an individual future observation taking into account the uncertainties arising from all the regression parameters into the total uncertainty ( Rawlings *et al.*, 1998).

For each CHN run, we estimated *σ _M_* as stated in Altman (2000).

$$\;\sigma_{M_{ik}}=t_{1-\alpha /2}*\sigma_{\text{res}}\sqrt{1+\frac{1}{n_{S}}+\frac{{\left(x_{ik}-\bar{x}\right)}^{2}}{(n_{S}-1)s_{x}^{2}}},\;(14)$$

where *σ*_res_ represents the robust standard deviation of the residuals of carbon mass about the regression line, *x _ik_* is the instrument response ( Equation 1), $\bar{x}$ and *s _x_* are the mean and the standard deviation of *x _ik_*, *n _S_* is the number of standards used to fit the model, and *t*_1 *−α/*2_, is the value from the *t* distribution with *n _S_ −* 2 degrees of freedom and *α* equal to 0.32 corresponding to a 68% prediction interval.

The uncertainty of the regression model and the estimated masses depended on both the uncertainty of the weight of the standards and the sensitivity of the instrument to the carbon at the time when filters were processed. Therefore, *σ*_res_ and *σ _M_* varied among CHN runs. Two CHN runs had significantly higher *σ*_res_ and therefore higher uncertainties of the regression models and mass estimates, compared to the rest of the runs. We believe that the cause of the poorer performance of the regression analysis in these instances was a less accurate weighting of the standards, and not an instability or low sensitivity of the CHN analyser. The differences of the duplicate POC estimates derived from these regression curves, on average, were not larger than those derived from regression curves with lower uncertainties ( Figure 2). This means that there was no bias in the less accurate standards - they resulted in unbiased calibration coefficients, even though the random uncertainties around these coefficients were higher. Nevertheless, to avoid skewing our results, we did not include data derived from these two CHN runs when we determined our total experimental uncertainty ( Equation 11).

The uncertainties of carbon masses estimated for uPOC and aDOC using the regression models were propagated into the uncertainties of POC masses by applying Equation 5 to Equation 2:

$$\sigma_{M_{\text{P}}}=\sqrt{\sigma_{M_{\text{uPOC}}}^{2}+\sigma_{M_{\text{aDOC}}}^{2}-2\sigma_{M_{\text{uPOC}}}\sigma_{M_{\text{aDOC}}}r\;(M_{\text{uPOC}},M_{\text{aDOC}})},\;(15)$$

where *r*( *M*_uPOC_, *M*_aDOC_) is the correlation coefficient between *M*_uPOC_ and *M*_aDOC_.

Then, for each estimate of POC concentration, we calculated the contribution of this source of uncertainty to the total uncertainty of POC concentration, *σ _C_* ( *M* ):

$$\;\sigma_{C}(M)=\frac{\sigma_{M_{P}}}{V}.\;(16)$$

***2.5.3 Uncertainty due to sample handling during CHN analysis.*** The stages of acidification and drying of the filters may contaminate the samples, thus adding positive biases to the estimates of POC concentration and a higher variability between the duplicates ( King *et al.*, 1998).

Considering that the uPOC samples, their corresponding aDOC blanks, and the filters blanks were acidified in the same desiccator and dried together in the oven, the amount of contamination that they received should have been approximately equal. Thus, the effect of the possible contamination should have been removed from the estimates of POC during the subtraction of $M_{\mathrm{aDOC}}^{\text{*}}$ from $M_{\mathrm{uPOC}}^{\text{*}}$ in Equation 2. However, there was a degree of random uncertainty associated with the estimation of contamination as the average mass of carbon of the three acidified filter blanks for each desiccator, ${\bar{M}}_{ac_{j}},$ varied slightly.

The contribution of the uncertainty due to the handling of the filters to the total uncertainty, *σ _C_* ( *η*), can be calculated by applying the standard law of propagation of uncertainty to Equation 4:


$$\sigma_{C}(\eta )=\frac{1}{\text{V}}\sqrt{\sigma_{\eta_{1}}^{2}+\sigma_{\eta_{2}}^{2},}\;(17)$$

where *σ*_*η*_1__ and *σ*_*η*_2__ represents the uncertainties of contamination due to handling of uPOC and aDOC filters, respectively. These uncertainties were estimated as the standard error of the mean of the three corresponding estimates of acidified filter blanks, $\sigma_{{\bar{M}}_{ac}}.$

## 3 Results and discussion

### 3.1 Distribution of POC

**Figure 3.**
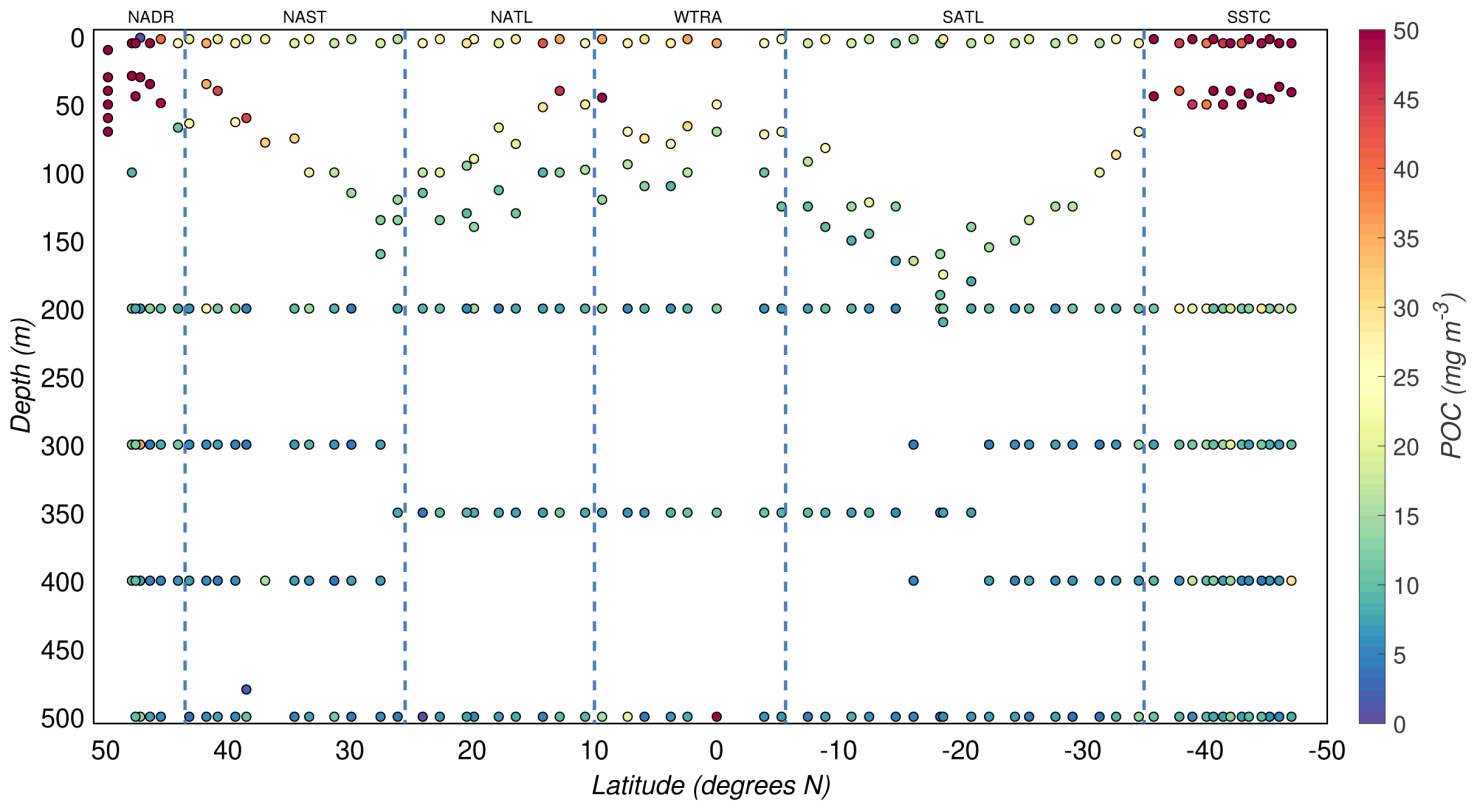
Depth-resolved distribution of particulate organic carbon (POC) concentration along the AMT-24 cruise. Borders of the sampled biogeographical provinces are marked by blue vertical lines.

The POC concentrations from the AMT-24 cruise were highly variable, ranging between 2 and 76 mg/m ^3^ ( Figure 3). The productive zone was characterised by relatively high POC concentrations, while low POC concentrations defined the mesopelagic zone. The overall median POC concentration from the productive zone was 18( *±*14) mg/m ^3^, whereas that from the mesopelagic zone was 7( *±*3) mg/m ^3^. We also observed latitudinal patterns of POC driven by the seasonality and differences between the biogeographical regimes sampled during the cruise ( Figure 1 and Figure 3). In the productive zone, the highest POC concentrations were found at temperate latitudes and around the equator, whereas the most oligotrophic provinces were characterised by lower POC concentrations ( Table 3). Such distribution in the POC concentration matched the typical latitudinal patterns encounter for the upper layer of the Atlantic ocean ( Poulton *et al.*, 2006; Rasse *et al.*, 2017; Wangersky, 1976). Particularly, high POC concentrations were found in the sub-surface (50 m) of the South Subtropical Convergence Zone. In this province, Poulton *et al.* (2006) found relatively high POC concentrations deeper than 200 m as a result of sinking particles from the euphotic zone of the SSTC. Overall, POC concentration below the productive zone was less variable throughout the transect, but the latitudinal pattern remained (see Table 3). Even though our POC concentrations are comparable to those published in the literature, a direct comparison is complicated due to differences in methodologies, sampling times, and regions. The range of POC concentration estimated from bottle samples in the Atlantic ocean by numerous researchers spans from 5 to 350 mg/m ^3^ ( Balch *et al.*, 2010; Banoub & Williams, 1972; Cetinić *et al.*, 2012; Gardner *et al.*, 1993; Gardner *et al.*, 2003; Gardner *et al.*, 2006; Graff *et al.*, 2015; Marra *et al.*, 1995; Menzel & Goering, 1966; Mishonov *et al.*, 2003; Poulton *et al.*, 2006; Stramska & Stramski, 2005; Stramski *et al.*, 2008; Wangersky, 1974; Wangersky, 1976). A limited number of studies present POC concentrations measured in the Atlantic ocean between 100 and 500 m and spanning from *∼*0 to 35 mg/m ^3^ ( Carlson *et al.*, 2000; Cetinić *et al.*, 2012; Menzel, 1967; Poulton *et al.*, 2006; Wangersky, 1974; Wangersky, 1976).

**Table 3.** Particulate organic carbon distribution presented for productive and mesopelagic zones across the sampled biogeographical provinces: the NorthAtlantic Drift Province (NADR), the North Atlantic Subtropical Gyral Province (NAST), the North Atlantic TropicalGyral Province (NATL), the Western Tropical Atlantic Province (WTRA), the South Atlantic Gyral Province (SATL), and the South Subtropical Convergence Province (SSTC). "Std" are robust standard deviations. Median and Std values are expressed in mg/m ^3^.

No	Province	Productive zone			Mesopelagic zone		
		Samples	Median	Std	Samples	Median	Std
1	NADR	25	33	31	16	10	9
2	NAST	36	17	7	33	7	3
3	NATL	36	15	8	18	8	2
4	WTRA	32	19	10	16	9	6
5	SATL	57	14	5	44	7	2
6	SSTC	39	46	16	39	9	3
7	ALL	225	17	14	167	7	4

### 3.2 Detection limits

To determine the limit of detection of our technique we used the approach recommended by The International Union of Pure and Applied Chemistry (IUPAC) and The International Organization for Standardization (ISO) ( Analytical Methods Committee AMCTB No. 92, 2020). We calculated the detection limit, ( *L _D_*), by first computing the critical value, ( *L _C_*, Equation 18), which establishes the presence of the analyte (carbon in our case), and is defined as the minimum significant estimated value of an analytical result, which is used as to discriminate against background noise ( Currie, 1995):

$$L_{C}={\bar{x}}_{0}+s_{0}*t_{0.95;df,}\;(18)$$

where ${\bar{x}}_{0}$ and *s*_0_ are the mean and the standard deviation of a blank material free from carbon, in our case, the tin capsules. *t*_0.95; *df*_ is the one-tailed 95% quantile for Student’s *t* with degrees of freedom *df*, according to the number of values used to estimate ${\bar{x}}_{0}$ and *s*_0_.

Given *L _C_*, we estimated *L _D_*, which is defined as the lowest carbon mass that our analytical method is reliably capable of detecting 95% of the time:

$$L_{D}=L_{C}+s_{0}*t_{0.95;df}={\bar{x}}_{0}+2s_{0}*t_{0.95;df.}\;(19)$$

**Figure 4.**
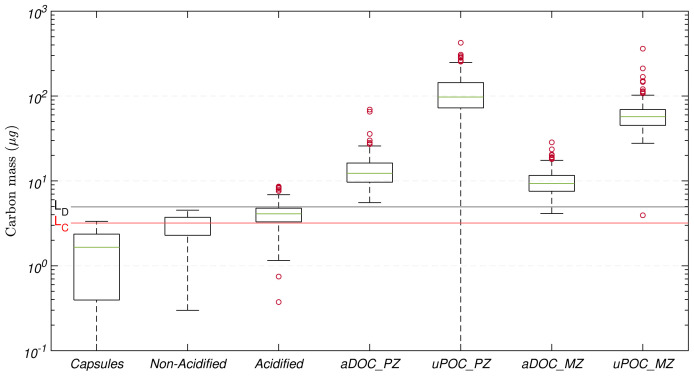
Distribution of carbon mass determined on tin capsules, non-acidified and acidified blank filters (GF/F) used for sample processing, and adsorbed dissolved organic carbon (aDOC) and uncorrected particulate organic carbon (uPOC) filters. In each element of the box plot, the central rectangle spans from the first quartile (25 *^th^* percentile) to the third quartile (75 *^th^* percentile). The green line inside each rectangle shows the median value and whiskers below and above the box show the locations of the 5 *^th^* and the 95 *^th^* percentile, respectively. Red circles represent outliers. Red and black horizontal lines represent the critical value ( *L _C_*) and detection limit ( *L _D_*), respectively.

The median of the carbon mass from all the tin capsules used in the 16 CHN runs was 1.7( *±*1.3) *µ*g. Thus, the estimated *L _C_* and *L _D_* were 3.2 and 4.9 *µ*g C, respectively ( Figure 4). The vast majority of mesopelagic aDOC filters collected and analysed during this study were above *L _D_*, with just four filters fell between the estimated *L _D_*.

### 3.3 Correction for biases

We corrected additional sources of bias in the estimates of POC concentration by subtracting aDOC blank measurements from the corresponding uPOC measurements (see Section 2.3 and Equation 2). Since the carbon mass determined on an aDOC blank includes the mass of the adsorbed DOC, the carbon masses detected on empty tin capsules, clean GF/F filters and any contamination occurring during filter acidification and handling, it represents the cumulative bias for which the carbon mass on a corresponding uPOC filter needs to be corrected. Hence, to minimise biases introduced by any potential contamination and mass predicted by the calibration equation, we processed pairs of uPOC and aDOC filters together during sampling, acidification, handling, and processing stages, i.e., acidified in the same desiccator and analysed during the same CHN run.

The magnitude of the masses on these additional components in comparison with the magnitude of the masses of aDOC and uPOC filters are presented in Figure 4. The median of the carbon mass from all the tin capsules used in the 16 CHN runs was 1.7( *±*1.3) *µ*g, whilst the medians of the mass corresponding to non-acidified and acidified filter blanks were 3.2( *±*0.8) *µ*g and 4.1( *±*1.1) *µ*g, respectively, indicating that our method minimised contamination during acidification.

On average, the acidification and handling of the filters resulted in contamination of 1( *±*1) *µ*g. Nevertheless, when comparing corresponding sets of acidified and non-acidified filter blanks, the carbon masses from the acidified filters could be up to twice as large the amount of those from the non-acidified filters. Finally, the cumulative effect of all the biases and potential contamination that composed our aDOC blanks were 12( *±*4) *µ*g in the productive zone and 9( *±*3) *µ*g in the mesopelagic zone.

**Figure 5.**
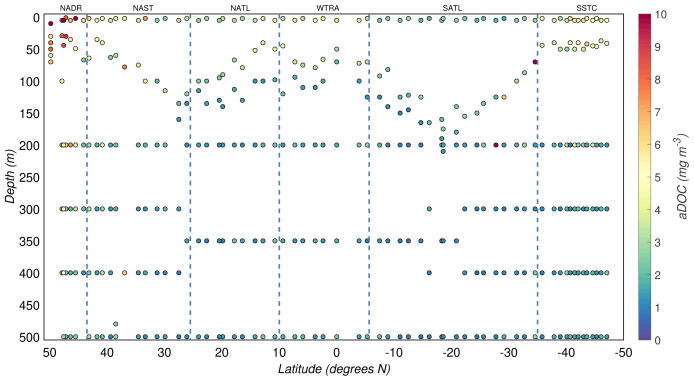
Depth-resolved distribution of adsorbed dissolved organic carbon (aDOC) estimates (mass of carbon on aDOC filters normalized as a function of volume and corrected for biases) along the AMT-24 cruise. Borders of the sampled biogeographical provinces are marked by blue vertical lines.

The range in carbon masses of our aDOC ( Figure 5) is comparable with those from the Atlantic ocean reported by Cetinić *et al.* (2012): their average mass of DOC adsorption was 10.9 *µ*g with 95% of the masses of their aDOC blanks within the range of 8.5 to 40.5 *µ*g. Also, our aDOC values are within the findings of Abdel-Moati (1990), who reported varying amounts of DOC adsorption between 9.2 and 15.0 *µ*g and 3.5 and 6.5 *µ*g for eutrophic and oligotrophic waters, respectively. The aDOC concentrations (carbon mass on aDOC filters normalized by volume and corrected for biases) in the productive zone had a median value of 2.2( *±*1.3) mg/m ^3^, and 1.0( *±*0.5) mg/m ^3^ in the mesopelagic zone. The distribution of theseconcentrations was correlated with POC concentrations ( *r* = 0.7, Figure 8 and Figure 9). This correlation was particularly strong in the productive zone ( *r* = 0.87 vs. *r* = 0.38 in the mesopelagic) and in regions characterized by high surface concentrations of POC: NADR ( *r* = 0.8) and SSTC ( *r* = 0.9).

Since the filtered volumes were adjusted according to the expected POC concentration, and all GF/F filters were from a single manufacturer and treated identically during the cruise, the aDOC concentration should be relatively constant across the samples and not correlated with POC concentration. However, POC could be present on the aDOC filters, increasing the aDOC concentration due to fragmentation of particles through uPOC filters ( Bishop & Edmond, 1976), perhaps due to the increased pressure differential caused by the two stacked filters ( IOCCG POC Protocols). This fragmentation of larger particles from the uPOC filters into smaller particles has been observed ( Wangersky, 1974) to contaminate aDOC filters and, perhaps, causing the higher aDOC values found in our study. For instance, Banoub & Williams (1972) filtered multiple samples of seawater through four stacked GF/C filters and found particles as evidence of contamination on the second filter from top to bottom. Abdel-Moati (1990) carried out a similar experiment with similar conclusions, suggesting the sum of masses from the first two filters should be used as a POC mass, while using the third filter, from top to bottom, as a true blank for aDOC. Cetinić *et al.* (2012) also pointed to the high variability of their aDOC values and potential contamination from the overlying uPOC filters. Finally, we note that when cells break, the DOC they contain is released in the environment and not retained on filters, potentially introducing biases in the POC determination.

If a higher than usual aDOC value is due to the contamination of an aDOC filter with particles filtered through the uPOC filter, particle loss from the uPOC filters should be higher in productive areas, thus explaining the observed strong positive correlation between POC and aDOC concentrations ( Figure 9; see also Zhou *et al.*, 2016). Assuming that particles contaminated the aDOC filters, we expect that the typical mass of organic carbon adsorbed onto our GF/F filters should be better represented by the aDOC measurements at depth, where particles are less abundant. Thus, we estimated the loss of particles from uPOC to aDOC filters by subtracting from the carbon mass of all aDOC blanks (corrected for biases), the median carbon mass of aDOC filters collected in the mesopelagic zone (≥200 m). Then, we added this difference to our POC masses and found that POC concentrations increased by 3( *±*4)% in the productive zone and by 0( *±*5)% in the mesopelagic zone.

We cannot prove which mechanism determined the correlation between aDOC and uPOC concentrations. Nonetheless, the carbon mass ratio ( $M_{\mathrm{aDOC}}^{\text{*}}$ / $M_{\mathrm{uPOC}}^{\text{*}}$ , corrected for biases, ranged from 3.5 to 58.2% with medians of 9.4( *±*2.2)% and 12.0( *±*3.4)% in the productive zone and the mesopelagic zone, respectively, indicating that DOC adsorption was important in the open ocean.

An additional reason that aDOC and POC concentrations showed a positive correlation pattern could be because DOC and POC have a similar decreasing pattern as a function of depth in the open ocean ( Dai *et al.*, 2009).

### 3.4 Relative and total experimental uncertainties

**Figure 6.**
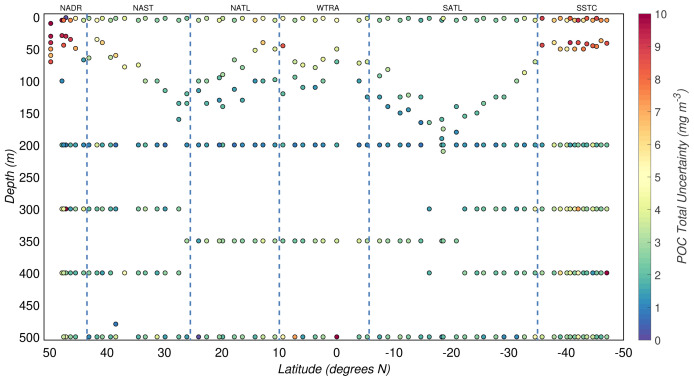
Depth-resolved distribution of the total uncertainty associated with the estimates of particulate organic carbon (POC) concentration derived during the AMT-24 cruise. Borders of the sampled biogeographical provinces are marked by blue vertical lines.

The relative experimental uncertainty of POC concentrations, *σ _d _r__*, ( Equation 9), was on average ∼14% and ∼35% in the productive zone and the mesopelagic zone, respectively. Higher *σ _d _r__* were estimated for the mesopelagic zone where POC concentrations were lower and biases might have had a greater effect on the estimates. The resulting total experimental uncertainties of the estimates of POC concentration ( *σ _C_*) are shown in Figure 6. The median of *σ _C_* for the productive and mesopelagic zones were 2.5( *±*1.2) mg/m ^3^ and 2.6( *±*0.6) mg/m ^3^, respectively.

### 3.5 Uncertainty budget

The modeled uncertainty for POC is presented in Table 4 for each component, and compared to the estimated total experimental uncertainty of POC derived from the duplicate measurements. The uncertainty in volumetric measurements *σ _C_* ( *V*) typically contributed about 1% to the total uncertainty of POC concentrations in the mesopelagic zone. However, since the volume of water filtered in the productive zone was smaller, the contribution of volumetric uncertainties was greater (∼2%). Overall, the contribution of this source of uncertainty was insignificant, except when POC concentration was particularly high and, as a consequence, the volume of the water sample was ∼2 L.

**Table 4.** Uncertainty budget presenting the contributions of each source of uncertainty that we could quantify, *σ _C_* ( *x _i_*), relative to the total experimental uncertainty of particulate organic carbon, *σ _C_*. Median values are given for the productive and mesopelagic zones.

No	Source	*σ _C_* ( *x _i_*) */σ _C_*	
		Productive zone	Mesopelagic zone
1	Volume	0.02	0.01
2	Calibration	0.17	0.12
3	Laboratory sample handling	0.03	0.02
4	Unquantified	0.78	0.85

**Figure 7.**
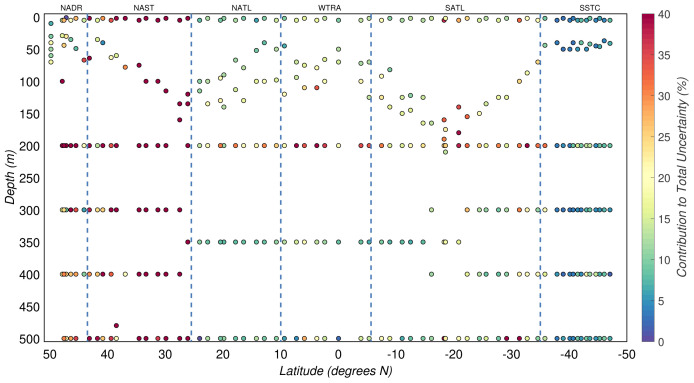
Portion of the total uncertainty of particulate organic carbon concentration that can be explained by the uncertainty of regression analysis. Borders of the sampled biogeographical provinces are marked by blue vertical lines.

The uncertainty due to the calibration equation *σ _C_* ( *M _P_*), after excluding the unstable CHN runs, explained a median of 16( *±*20)% of the total experimental uncertainty of POC concentrations ( Figure 7). Since unstable CHN runs were characterised by greater residual errors in the regression analysis, their contribution to the total experimental uncertainty was significantly higher with a median of 65( *±*36)%.

Our acidification method and handling during the CHN analyses allowed us to minimise the effect of contamination of POC estimates. The median contribution of this source of uncertainty, *σ _C _P__* ( *η*), in productive waters was 3( *±*3)%, while in the mesopelagic zone attained 2( *±*2)%.

**Figure 8.**
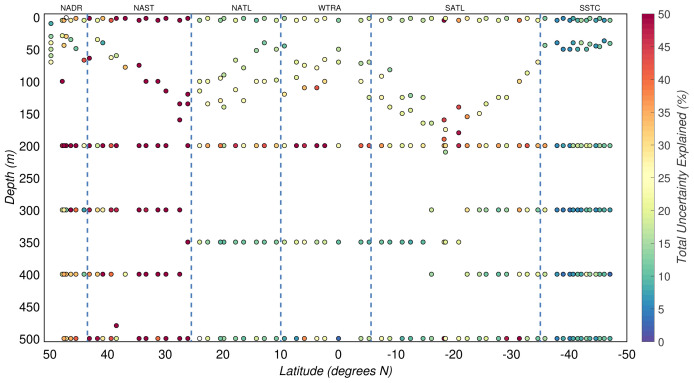
Portion of the total uncertainty of particulate organic carbon concentration that can be explained by the uncertainty budget. Borders of the sampled biogeographical provinces are marked by blue vertical lines.

Overall, the three sources of uncertainty described above explained only 21( *±*22)% of the total experimental uncertainty in POC. Thus, other sources of uncertainty must be responsible for the relatively large and unexplained part of the estimated experimental uncertainty ( Figure 8).

**Figure 9.**
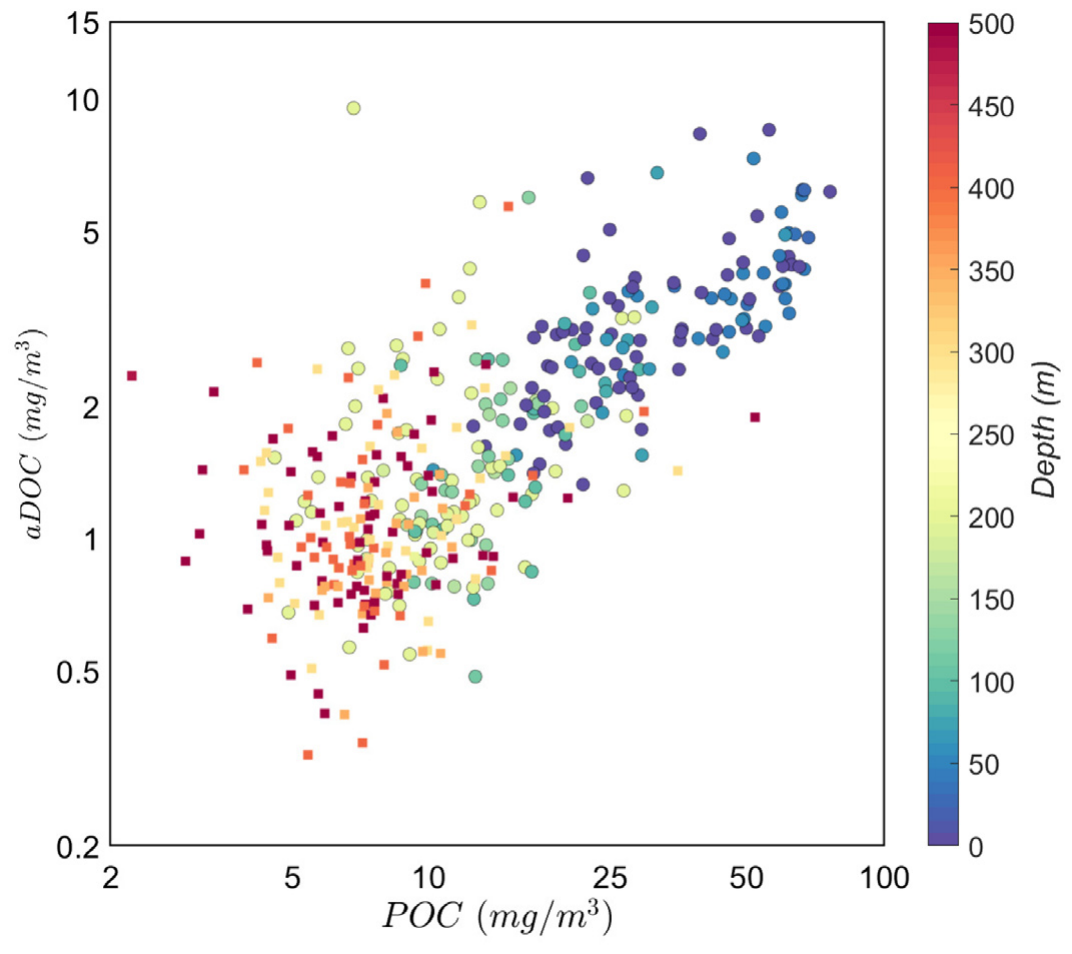
Correlation between aDOC and POC values. Colours refer to sample depths. Squares represent mesopelagic samples (MZ, r = 0.38). Circles represent samples in the productive zone (PZ, r = 0.87).

### 3.6 Missing sources of uncertainty

In this section we discuss potential sources of uncertainty that could explain the missing part of the uncertainty budget.

***3.6.1 Rare particles.*** Patchiness is ubiquitous in the ocean, ranging from microscale thin layers in the water column, to sub-mesoscale and mesoscale fronts, and could have contributed to our total experimental uncertainties in POC. For instance, Bochdansky *et al.* (2016) analysed the dynamics and abundances of particles at depth using a custom-made digital inline holographic microscope. They found that the concentration of patchy marine snow (large particles >500 *µ*m) was 100 times higher than expected in comparison with the concentration of smaller particles. Additionally, Ohman *et al.* (2012) analysed the concentration and vertical distribution of suspended particulate matter and mesozooplankton at a deep-water front in the California Current System using a high resolution digital camera system. They observed that the front had a different composition of particulate matter, and it was a zone of higher marine snow particles where the volume of all size fractions of suspended particulate matter, especially organic aggregates, increased several times in comparison with the surrounding seawater. Therefore, despite mixing samples in carboys before dispensing them to the filtration bottles, some of our duplicate filters might have captured different types of particles, i.e. rare large aggregates or (invisible) zooplankton might have appeared only on one of the two duplicate filters.

Furthermore, Wangersky (1974); Wangersky (1976) found that a water mass in the open ocean had a homogeneous background of POC concentration, upon which occasional small patches with up to five times POC concentration were superimposed. He reported uncertainty of a single POC estimate derived from replicate 5-litre samples to be equal to 2.5( *±*1) mg/m ^3^.

Our median total uncertainty for the mesopelagic region of 3( *±*1) mg/m ^3^ is comparable to these findings, suggesting that samples might have been drawn from water masses of similarly spatially variable POC concentrations.

Further experiments are required to better understand the natural heterogeneity and small-scale patchiness of seawater using Niskin bottles. Since the collection of intact and flocculent marine snow particles using bottle samplers is highly challenging and time-consuming ( Bochdansky *et al*., 2016), the oceanographic community would benefit from analysing the natural heterogeneity of suspended particles and the effect of patchiness on estimates of POC concentration and their uncertainties using optical observations from instruments such as the Underwater Vision Profiler or holographic cameras which have the advantage of displaying details of the *in*- *situ* vertical distribution of particles across a range of sizes ( Bochdansky *et al*., 2016; Ohman *et al*., 2012).

***3.6.2 Contamination during filtration.*** The filtration system employed during this study was an open-funnel filtration set. This set up might have increased the risk of contamination by exposing the samples to particles rich in carbon (e.g., dust, ashes, ship’s engine exhaust), during sampling from the Niskin bottles and during filtration in the laboratory ( Gardner *et al*., 2003);( IOCCG POC Sampling and Measurement Protocols).

We consider that mainly uPOC filters would have been contaminated in such a way because they were exposed to the laboratory atmosphere for longer than the underlying aDOC filters. Assuming that the uPOC filters were contaminated during filtration, their duplicate filters might have received a similar amount of contamination. Thus, atmospheric contamination of uPOC filters would result in greater differences between uPOC duplicates compared to the differences between less contaminated aDOC duplicates. Indeed, the median of the absolute differences between duplicate uPOC concentrations was 2( *±*2) mg/m ^3^, which is higher than the median of the absolute difference between duplicate aDOC concentrations of 0.2( *±*0.4) mg/m ^3^. This result, however, is also consistent with our previous finding that the differences between duplicate concentrations depend on POC concentrations (see Figure 2). As a consequence, we do not have enough grounds to state that differences between duplicate uPOC concentrations are higher than the differences between duplicate aDOC concentrations because of contamination of uPOC filters.

The longer a filter is exposed to the laboratory atmosphere, the more contamination it should receive. The duration of filter exposure to the laboratory atmosphere depends on the volume of seawater filtered through the filter. To further investigate contamination of uPOC filters, we analysed how differences between duplicate uPOC concentrations depended on differences between volumes of seawater filtered through two duplicate filters and found no significant correlation.

Thus, there was no evidence to suggest that the time of exposure of filters to the atmosphere was the cause of disagreement between duplicate estimates. However, analysing duplicates to determine this source of contamination might not be sufficient as duplicate estimates could have been affected by other biases or contaminants that mask a single source of contamination. Consequently, with the available data, we cannot quantify this source of contamination and its uncertainty.

For future experiments, especially when using an open-funnel setup, filtering Milli-Q water through additional blank GF/F filters may be used to quantify contamination from the laboratory atmosphere. Even better, to minimise contamination from airborne particles, it would be advisable to filter samples under a laminar flow hood or by employing a closed filtration system ( Cetinić *et al*., 2012).

***3.6.3 Storage of samples.*** Freezing and storing of sample filters might have also introduced some contamination. Published values suggest that the average mass of unused GF/F filters from a cruise may range from 3( *±*10) *µ*g to 10( *±*5) *µ*g ( Cetinić *et al*., 2012; Menzel, 1966; Stramski *et al*., 2008; Wangersky, 1974). The difference between our non-acidified (3 *±*1 *µ*g) filter blanks and these published values might indicate that the contamination during filter storage could amount to between 0 and 7 *µ*g. However, assuming homogeneous contamination of uPOC and aDOC filters, we would expect that this contamination would be accounted for when aDOC is subtracted from uPOC ( Equation 2). To quantify uncertainties due to filter storage, we recommend preserving multiple unused GF/F filter blanks along with the samples for post-cruise analysis.

***3.6.4 Collection of samples.*** Uncertainties can also be introduced by different operators. In our case, samples were collected by two operators, whereby one operator systematically collected samples from pre-dawn casts, while the other from noon casts. Thus, we thought that analysing duplicates pairs collected during pre-dawn and noon casts separately might give us an insight into this source of uncertainty. Due to constraints in the water budget, pre-dawn duplicates were collected from quasirandom depths, while 25 out of 28 duplicate pairs collected during noon time represented deep waters ( *≥*400 m). For pre-dawn duplicates, the median POC concentration and the median absolute differences of duplicate POC concentrations were 9( *±*9) and 1( *±*2)mg/m ^3^, respectively, while for noon duplicates were 9( *±*5) and 2( *±*2) mg/m ^3^, respectively, suggesting that there was no statistical difference between these medians in the two groups of duplicates. If we take into consideration that the majority of noon duplicates were collected from deep waters ( *≥*400 m), pre-dawn duplicates seem to be slightly more precise than noon duplicates with medians of the relative differences of duplicates collected from deep waters of 19( *±*10)% and 19( *±*31)% for pre-dawn and noon duplicates, respectively. However, there is no evidence that samples collected by different operators are biased by the operators themselves rather than by varying composition of particles at different depths. Even though the higher uncertainties that we found in the mesopelagic zone might be partially explained by the varying precision of duplicates collected by the two operators from pre-dawn and noon casts, we cannot quantify the bias and the uncertainty introduced by each operator.

***3.6.5 Uncertainty model.*** The uncertainty model we employed in this study ( Equation 11) was based on the empirical relationship we observed between duplicate differences and POC concentrations ( Figure 2). Admittedly, this model is likely an approximation of the experimental uncertainty of our POC measurements. To improve the model (e.g., by adding a constant offset to it), we would need to better understand the role that each source of uncertainty plays in the total uncertainty of POC concentrations and to reconcile experimental and theoretical estimates of the POC uncertainty. These additional steps would ultimately allow us to then understand the extent to which each source of uncertainty is either a multiplicative or an additive term to the total uncertainty as POC, as the POC concentration in the ocean varies. Further dedicated experiments and analyses would be needed to achieve this deeper understanding.

### 3.7 aDOC blanks

Even though the double-filter technique employed in this study significantly increases filtration times, this procedure allowed us to collect a sample blank (i.e., aDOC filter) for each uPOC filter. Therefore, it is interesting to investigate how the uncertainty in the final POC concentrations would vary if fewer or no aDOC blanks were collected.

Some researchers avoid collecting aDOC blanks, under the assumption that by maximising the filtered volumes of seawater, uncertainties related to aDOC could be minimised ( Stramski *et al*., 2008). Since we collected multiple types of blanks (i.e., aDOC, non-acidified, and acidified), we can test this assumption under different scenarios.

First, uPOC concentrations were higher than POC concentrations by about 13( *±*7)% in the productive zone and 19( *±*11)% in the mesopelagic zone. Second, by subtracting the median carbon mass of non-acidified filters (i.e., clean GF/F filter blank) from the uPOC carbon mass, we obtained uPOC concentrations (corrected from clean GF/F filter blank) that are greater than our POC concentrations by 9( *±*6)% in the productive zone and 12( *±*9)% in the mesopelagic zone. Finally, by subtracting the median carbon mass of the acidified filters from the uPOC carbon mass, the uPOC concentrations (corrected from acidified GF/F filter blank) are larger than our original POC concentrations by 8( *±*6)% in the productive zone and 10( *±*8)% in the mesopelagic zone. Thus, by not correcting for aDOC blanks, we would have introduced positive biases in POC concentrations of the order of 10–20%, even if we filtered up to 8 liters of water. It is important to realise that these results depend on the relative amount of adsorbed DOC and POC present on the filters and therefore on the volumes of water filtered and the POC concentration. One must be careful when extrapolating our conclusions to POC values determined from different sample volumes and different ocean regions.

To reduce filtration time, one could collect fewer aDOC blanks (e.g., only one deep sample per station). To quantify the potential uncertainty introduced by this method, we corrected our uPOC estimates using a single value of aDOC blank, which was determined from the median of the aDOC blanks from deep ( *≥*200 m) stations. The resulting POC concentrations were 3( *±*4)% and 0( *±*6)% higher than the original concentrations in the productive and the mesopelagic zones, respectively. Thus, by using fewer aDOC blanks, one could significantly reduce the bias generated when not using an aDOC blank. Overall, the above exercises can guide quantitatively how many (if at all) aDOC blanks to collect, based on the level of uncertainty that one is willing to accept.

Finally, an alternative method to decrease filtration times could be to collect aDOC samples by filtering smaller amounts of water, by collecting aDOC samples separately from the uPOC ones. For example, based on an analysis of various coastal and open-ocean samples Novak *et al*. (2018) suggested that GF/F filters are saturated with DOC after about 0.6 liters of sample water have been filtered. Even by adopting a conservative approach and doubling this suggested DOC saturation volume one could significantly decrease filtration times at sea, while ensuring that POC values are corrected for the adsorbed DOC.

### 3.8 The need for a POC reference material

The accuracy of oceanographic chemical analyses is typically assessed by measuring consensus or certified reference materials (CRM). Unfortunately, at present no such CRM has been selected by the oceanographic community for POC analyses ( IOCCG POC Sampling and Measurement Protocols). CRMs exist that potentially might be used to represent organic matter in the ocean, e.g., NIST Buffalo River Sediment RM 8704 (National Institute of Standards and Technology), but their precise bio-organic elemental composition has not been determined, which prevents one from assessing how representative they are of open-ocean pelagic particulate matter ( National Research Council, 2002). In addition, these CRMs are mainly comprised of marine sediments, rich in aluminosilicates and quartz, but with no pelagic opal and carbonate matrices, thus misrepresenting the complex matrix associated with open-ocean pelagic POC samples and potentially introducing artefacts in the accuracy assessment ( National Research Council, 2002). The Committee on Reference Materials for Ocean Science of the US National Research Council recommended that a consensus or certified reference material for POC representative of open-ocean particulate matter could be obtained by mixing cultures from a diatom, a dinoflagellate, and a coccolithophore ( National Research Council, 2002). Unfortunately, as of today, no CRM for POC analyses has been developed and, as a consequence, POC analyses are difficult to compare over time, among groups, and when different analytical protocols are used. Future work to improve POC determinations should focus on agreeing upon and producing a certified or consensus reference material. Finally, intercomparison exercises are also needed to minimise uncertainties arising from all sample collection and processing steps before the CHN analysis. Dedicated funding and an international effort are needed to achieve these two crucial objectives.

## 4 Conclusions

In this study, we modified the standard protocol used to determine POC concentration ( Knap *et al*., 1996; Martin *et al*., 1993) by: sampling different volumes of water in accordance to the expected in-situ POC concentrations; adopting different types of blanks, i.e., aDOC filter blanks and acidified and non-acidified GF/F filter blanks to quantify the dissolved organic carbon and any contamination (biases); and collecting and analysing duplicate samples. This methodology enabled us to quantify experimental uncertainties of our POC concentrations. Moreover, the various filters used as blanks allowed us to discuss several sources of uncertainty that contributed to the discrepancies between the duplicate estimates.

We found that the total experimental uncertainty of our POC estimates varied with depth and with POC concentration and was ~14% and ~35% in the productive and in the mesopelagic zones, respectively. However, the quantification of all the different sources of uncertainty associated with POC concentrations was impossible with our measurements, and we could explain only ~21% of the total experimental POC uncertainty we observed. Further work is required to identify the unexplained portion of the uncertainty.

Nevertheless, we moved one step forward in understanding uncertainties of bottle-derived POC estimates. The analysis of duplicates and of the various blanks presented in this paper improved our understanding of the limitations of the methods and of some of the stages of sample collection and processing that contributed to the variability of our results. Further experiments would be required to fully understand the uncertainty budget of POC estimates. This understanding would allow us to concentrate our effort on those parts of the methodology that are more prone to introduce uncertainties and to develop a better-informed protocol to foster comparability of POC estimates across different studies.

## Data availability

### Underlying data

Published Data Library (PDL), British Oceanographic Data Centre (BODC): AMT24 (JR20140922/JR303) Particulate organic carbon (POC) measurements from CTD bottles. http://doi.org/10/fzw5 ( Dall’Olmo *et al*., 2021).

This project contains the following underlying data:

- README.txt. (contains important information that is commonly required to understand the following files or spreadsheets deposited in CSV format.)
- Standards.csv. (Contains information regarding standards used to calibrate the CHN analyser, see README.TXT)
- Capsules.csv. (Contains information regarding empty tin capsules used to estimate uncertainties related to the sample handling in the lab, see README.TXT)
- NonAcidifiedFilters.csv. (Contains information regarding non-acidified filter blanks used to estimate uncertainties related to the sample handling in the lab, see README.TXT)
- AcidifiedFilters.csv. (Contains information regarding acidified filter blanks used to estimate uncertainties related to the sample handling in the lab, see README.TXT)
- aDOCFilters.csv. (Contains information regarding aDOC filters used to determine POC concentrations, see README.TXT)
- uPOCFilters.csv. (Contains information regarding uPOC filters used to determine POC concentrations, see README.TXT)
- DuplicateaDOC.csv. (Contains information regarding duplicate aDOC filters used to estimate total experimental uncertainties, see README.TXT)
- DuplicateuPOC.csv. (Contains information regarding duplicate uPOC filters used to estimate total experimental uncertainties, see README.TXT)
- POC.csv. (Contains overall information regarding POC concentrations, including nominal depth, amount of seawater filtered for each filter, geographic coordinates, date and time of collection, POC mass and POC concentration of each sample, see README.TXT)

This dataset is available under the terms of the UK Open Government Licence version 1.0 for public sector information. This licence governs access to and use of Open Data supplied by the Natural Environment Research Council (NERC).

## Software availability

- Source code available from: https://github.com/pstrubinger/Uncertainties-of-particulate-organic-carbon-concentrations-in-the-mesopelagic-zone-of-the-Atlantic-o/tree/v1.0.0
- Archived source code at time of publication: https://doi.org/10.5281/zenodo.4630974 ( pstrubinger, 2021)
- License: GNU General Public License, version 3 (GPL-3.0)
