# Supplementary material for: Uncertainties of particulate organic carbon concentrations in the mesopelagic zone of the Atlantic ocean
Source: Open Res Eur. 2022 Jul 29;1:43. Originally published 2021 Apr 23. [Version 3] doi: 10.12688/openreseurope.13395.3 (PMC10446083; doi:10.12688/openreseurope.13395.3)
Supplement: Peer review history

# Peer review: Reviewer response for version 3

**DOI:** 10.21956/openreseurope.16202.r32039

**Published:** 2023-07-28

**Authors:** Hendrik Grotheer

**Affiliations:** 1 Alfred Wegener Institute Helmholtz Centre for Polar and Marine Research, Bremerhaven, Germany

Review of:

 “Uncertainties of particulate organic carbon concentrations in the mesopelagic zone of the Atlantic ocean” Manuscript by Paul Strubinger Sandoval et al.

 This manuscript describes the assessment of uncertainties associated with the quantification of particulate organic carbon concentrations in the ocean. POC is an important part of the marine carbon pump and its fade during sinking from the surface ocean to the sea floor controls the efficiency of long-term carbon sequestration.

 Reliable concentration measurements are a requirement for trustworthy interpretations of the data. Obtaining these concentrations is analytically challenged by low POC concentrations in the water column, high analytical blanks associated with sampling and sample processing, and patchiness of POC concentrations. As a result, true uncertainties of POC concentrations are hard to be determined but need to be assessed to provide confidence in the data.

 The authors provide a reproducible workflow, including replicate analysis and mathematical modeling, to assess the uncertainty of POC concentrations. Further they attempt to partition the overall uncertainties to the separate working steps required to obtain the concentration, to identify the factors influencing the uncertainties.

 Even though the experimental design (especially sample collection at sea) does not appear to be tailored for this study, the authors provide a valuable framework for the community. With some optimizations, this work can be used as a basis to assess not just the true overall uncertainty of POC concentrations but also for isotope studies (d ^13^C and D ^14^C).

 Considering the revisions made to this manuscript based on the previous reviewer reports I support its publication.

Is the rationale for developing the new method (or application) clearly explained?

Yes

Is the description of the method technically sound?

Yes

Are the conclusions about the method and its performance adequately supported by the findings presented in the article?

Yes

If any results are presented, are all the source data underlying the results available to ensure full reproducibility?

Yes

Are sufficient details provided to allow replication of the method development and its use by others?

Yes

Reviewer Expertise:

Chemical Oceanography, Carbon Pump, Analytical Chemistry, Organic Geochemistry, Isotope Geochemistry

I confirm that I have read this submission and believe that I have an appropriate level of expertise to confirm that it is of an acceptable scientific standard.

# Peer review: Reviewer response for version 3

**DOI:** 10.21956/openreseurope.16202.r29876

**Published:** 2022-08-10

**Authors:** Anna Belcher

**Affiliations:** 1 Ecosystems Team, British Antarctic Survey, Cambridge, UK

Dear Authors,

 Thank you for your responses and the revisions you have made based on the comments of each reviewer.

 The work you present is of value to the scientific community and I endorse its indexing.

Is the rationale for developing the new method (or application) clearly explained?

Partly

Is the description of the method technically sound?

Partly

Are the conclusions about the method and its performance adequately supported by the findings presented in the article?

Partly

If any results are presented, are all the source data underlying the results available to ensure full reproducibility?

Partly

Are sufficient details provided to allow replication of the method development and its use by others?

Partly

Reviewer Expertise:

NA

I confirm that I have read this submission and believe that I have an appropriate level of expertise to confirm that it is of an acceptable scientific standard.

# Peer review: Reviewer response for version 2

**DOI:** 10.21956/openreseurope.15193.r27551

**Published:** 2021-11-01

**Authors:** Anna Belcher

**Affiliations:** 1 Ecosystems Team, British Antarctic Survey, Cambridge, UK

The authors have added in extra explanation and detail which is great to see. The paper provides data that will benefit the scientific community and presents uncertainties in a range of scenarios to allow others to adopt the necessary methods for the level of uncertainty they are willing to accept. It is good that the authors highlight the importance of correcting for adsorbed DOC whilst also highlighting the potential for contamination of POC on the aDOC lower filter. Their work suggests that having sufficient mesopelagic samples, where POC concentrations are lower to help estimate this contamination. They also present a useful investigation on the use of aDOC blanks, with an appreciation for what is practical at sea , having extra information on the possible bias if fewer aDOC blanks are taken.

 Considering that POC concentrations can be very patchy, as discussed by the authors, I think it would be good to highlight this in the conclusions, as in some areas of the ocean in particular, this patchiness, and resultant difference between replicates will be far far larger than the methodological uncertainties here. Perhaps also in section 3.6.1 the authors could provide their recommendations/suggestions for how to take into account this patchiness, in terms of number of replicates etc. Or at least a mention that this needs to be considered when deciding on the sampling area and number and location of replicates to characterise a region etc.

 I think the paper makes a contribution to the field and that the authors have explained the limitations of their works and uncertainties that they cannot quantify.

Is the rationale for developing the new method (or application) clearly explained?

Partly

Is the description of the method technically sound?

Partly

Are the conclusions about the method and its performance adequately supported by the findings presented in the article?

Partly

If any results are presented, are all the source data underlying the results available to ensure full reproducibility?

Partly

Are sufficient details provided to allow replication of the method development and its use by others?

Partly

Reviewer Expertise:

NA

I confirm that I have read this submission and believe that I have an appropriate level of expertise to confirm that it is of an acceptable scientific standard, however I have significant reservations, as outlined above.

# Response

**Published:** 2022-07-20

**Authors:** Giorgio Dall'Olmo

We thank Dr. Belcher for the time invested in re-reviewing our manuscript. Below we present a point-by-point response to all her comments/suggestions. (Reviewer comments in italics)

*The authors have added in extra explanation and detail which is great to see. The paper provides data that will benefit the scientific community and presents uncertainties in a range of scenarios to allow others to adopt the necessary methods for the level of uncertainty they are willing to accept. It is good that the authors highlight the importance of correcting for adsorbed DOC whilst also highlighting the potential for contamination of POC on the aDOC lower filter. Their work suggests that having sufficient mesopelagic samples, where POC concentrations are lower to help estimate this contamination. They also present a useful investigation on the use of aDOC blanks, with an appreciation for what is practical at sea, having extra information on the possible bias if fewer aDOC blanks are taken.*

*Considering that POC concentrations can be very patchy, as discussed by the authors, I think it would be good to highlight this in the conclusions, as in some areas of the ocean in particular, this patchiness, and resultant difference between replicates will be far far larger than the methodological uncertainties here.*

 RESPONSE: We have discussed particle patchiness at length in Section 3.6.1. Yet, we do not have evidence that leads us to believe that patchiness was the main source of the missing uncertainty that we have documented. Therefore, we do not feel it is justified to specifically mention this in the conclusions.

 ACTION: None taken.  

*Perhaps also in section 3.6.1 the authors could provide their recommendations/suggestions for how to take into account this patchiness, in terms of number of replicates etc. Or at least a mention that this needs to be considered when deciding on the sampling area and number and location of replicates to characterise a region etc.*

 RESPONSE: It is very difficult to provide details of what exactly should be done in the future, because these details depend on the particle patchiness in each specific water mass.

 ACTION: We have therefore only mentioned that this should be considered: “In future studies, when possible, these and other optical instruments should be used to quantify the patchiness of marine particles and its potential impacts on the uncertainty of the estimated POC concentrations. Such quantification could also inform the best strategies to collect discrete water samples in the presence of particle patchiness.”

# Peer review: Reviewer response for version 2

**DOI:** 10.21956/openreseurope.15193.r27737

**Published:** 2021-11-01

**Authors:** Frederic Planchon

**Affiliations:** 1 UMR 6539 LEMAR, CNRS, IRD, Ifremer, University of Brest, Plouzané, France

The manuscript presents an interesting and detailed dataset on the occurrence of POC and associated uncertainties in the upper water column of the Atlantic Ocean. This subject is relevant since POC concentrations especially in the mesopelagic zone remain poorly documented at a global scale. In addition, and despite decades of POC measurements, large discrepancies remain between the different methodologies used in terms of sampling techniques, sample preparation, storage, analysis and data validation. The present work, which is a revised version based on the comments of two reviewers, is a further contribution.

 The main objectives of the work are to quantify the uncertainties related to POC determination based on two independent approaches, the first one with duplicate samples and the second one with a detailed uncertainty budget taking into account three distinct contributions (volume, analytical determination, sample processing). These two estimates of the overall precision of the method are compared and discussed. The most important results of this study are that the determination of POC in the upper water column remains imprecise, especially in the mesopelagic zone (up to 35% error at 68% confidence interval) and adsorption of DOC represents a major source of bias that requires to be considered when POC is obtained from small volume seawater samples. The latter result is not surprising and has already been documented in a number of studies and should be better underlined in the abstract. The former result (precision deduced from duplicate samples) is far less documented and represents a valuable contribution that deserves publication. Regarding the methodology for estimating the precision, the duplicate sample approach is not common and probably suffer from a statistical significance as already mentioned by one reviewer. However, and as mentioned by the authors, this represents a valuable compromise.

 As a general comment, the paper reads well and is relatively well illustrated. The material and method section describes in much detail the samples considered, the analytical determination and the statistical analysis applied to the data. However, some points need clarification and better organization. Results should not be included in this section as much as possible. For instance, Figure 2 is presented in section 2.4.1 and is not necessary for the total experimental uncertainty calculation since no correlation is assumed between duplicates and the total uncertainty is calculated essentially using the scaled relative difference. It would be more appropriate to present the lack of correlation in the result section to confirm that your calculation is appropriate. Another occurrence of a result presentation can be found p10 after equation 11. Table 2, which summarizes the different contributions, should also be moved to later in manuscript and not introduced in section 2.5. Similarly, the section 3.2 dealing with the detection limit should be moved to the method section, at least for the details of the calculation.

 Regarding the detection limits, you choose to apply a 95% confidence interval, which makes sense and is commonly adopted in analytical chemistry. Why all other uncertainties are at 68% (1 sigma)?

 Still on the Ld and Lc values and as it can be deduced from Fig 4. It appears that all procedural blanks are below the Ld, only field blanks (aDOC) and samples (uPOC filters) are above. Does it make sense to report a C quantity that is below the Ld? It could even be worse if you considered the quantification limit.

 Regarding the total experimental uncertainty (section 2.4.1), I do not understand why it is necessary to apply a propagation law for estimating the precision (Eq. 7, 8, 9). Eq 10 related to the scaled relative difference of duplicates, which is actually your estimator of the relative precision, does not imply such a calculation. Absolute precision can be easily deduced by considering the average POC concentration of each duplicate. Still on this parameter (σΔr) and as mentioned in Hyslop and White (2009), it could be worth checking that your scaled relative differences are normally distributed. In that studies, three different estimators of the precision have been used including the root mean square (RSM), the scaled mean absolute difference (MAD) and the percentiles approach. Why did you choose the percentile precision?

 Another issue concerns the section 2.5.2 (uncertainty in mass predicted by the calibration equation), which is essentially the instrumental standard deviation. I do not understand why you include σMp in this section, which refers to the correction of total POC mass with the corresponding DOC mass. This parameter is not related to the calibration equation and represent a post-analysis correction made possible with your sampling methodology. I think it could be interesting to consider separately these two components (instrument precision and precision on the corrected POC quantity by the DOC quantity). At this point it is not clear what the values reported in table 2 and associated with the calibration correspond to, instrument precision or precision on the corrected mass.

 Still on the uncertainty budget in the section 2.5.3 (uncertainty due to sample handling during CHN analysis), the proposed calculation based on the three acidified filter blanks does not only correspond to contamination due to the acidification step but includes also the contribution from the filter. In Eq 17, I do not think it is necessary to introduce two distinct variables (ση1 and 2) since the two are equal and are estimated from the three acidified filter blanks. It could be interesting to consider separately these two distinct sources of uncertainty. Impact of acid fuming by considering the difference between non-acidified and acidified filter and the filter alone.

 Another remark on the impact of filter concerns their role in the analytical determination and especially on the sensitivity of the instrument. This is not taken into account in your dataset since your CHN standards were prepared without filters. Filters can affect the combustion process and as a consequence the sensitivity of the instrument (the slope of the calibration curve). Accordingly, it is highly recommended to tune the elemental analyzer with standards containing filters. In general, an increase in O2 gas is necessary to prevent partial combustion of standards and samples. Also, adding a filter to the standards allows for better reproduction of the sample matrix and provides a means for quantifying filter blanks through the regression analysis (b parameter). This procedure is required in C isotope studies for both concentration and isotopic corrections when samples are loaded on a filter.

 The result and discussion section is clearly written with abundant references to previous studies. I have only few concerns on this section. The first one is related to the description of the results. You present the median of your results with an associated uncertainty. Could you clarify how these uncertainties have been obtained (number of values and calculation)? My guess is that it corresponds to the standard deviation of your data. Looking at these uncertainties, they appear to be highly variable indicating a high dispersion of uncertainty data. I think this dispersion could be better considered and at least mentioned, especially when you compare the productive zone with the mesopelagic zone. For instance in section 3.7 when you compare uPOC with POC concentrations, I do not think that the difference between sampling zones is statistically different.

 Another point concerns the observed positive correlation between aDOC and POC concentrations (section 3.3 and figure 9). In addition to cells break and release of DOC, another hypothesis may concern the potential impact of small (<0.7 µm) particles associated with picophytoplankton (0.2-2µm in size) and small heterotrophs (free bacteria, archea, etc.). These organisms can be abundant in surface waters representing an important part of the biomass especially out of the bloom period.

 Detailed comments:

 Abstract L6-7: check sentences and change accuracy term with precision.

 Section 2.1: clarify the number of samples taken at each station, were they taken at the same cast? You mention 392 uPOC samples. if you collected 6 samples at 67 stations, this makes a total of 402 samples.

 Similarly, you mention duplicate samples were taken at each cast. You should have 67 duplicate samples and not 57. Please clarify

 Page 5, last paragraph: if filter blanks were placed in the dessicator they are obviously acidified. I would change to: process as filter sample and subjected to acid fuming in the dessicator.

 Page 6, third paragraph: correct your description of the sample analysis in the CHN. The samples are combusted in the oven (combustion chamber subjected to a dedicated temperature program), and then ashes fall in the ash trap, which progressively fills up. This is not a reaction tube. Then combustion gases are carried to the oxidation and reduction “tubes” (chambers) and then to the chromatographic column for separation and final detection.

 P6, paragraph 4-5: Theoretically, all standards measured require to be included in the calibration. The first set called “calibration standards” are generally used to check the linearity. For state of the art analytical sequence, linearity is checked also at the end of the sequence. Also, standards showing poor recovery (due to weighting error, bad analytical run) can be removed according to z-score.

 P6, paragraph 6: you often use the term robust throughout the manuscript (here for the fitting model), what does it mean? Does it differ from a classical linear fitting model?

 P6, bottom page: the two equations are not numbered and the definition of M star is not given. Furthermore, there is a missing term in these two equations corresponding to the C mass associated to the filter. The difference between acidified and non-acidified filter blanks corresponds only to the C added by the acidification process.

 P8, last paragraph: indicate the p value of the linear fits to explore the significance of the regression analysis.

 P9, figure 2: y axis of figure 2b is unitless.

 P10, section 2.5: an equation showing how you compute all uncertainty contributions would help the reader.

 P10, Table 2: to be clearer, add a column with the corresponding variables associated with each line. If this table is moved to the result section, please include the sd for each median value and adjust to significant digit.

 P14, last paragraph: indicate the p value of the linear fits and homogenize the significant digit.

 P17, table 4: same remarks as for table 2.

 P18, Figure 9: indicate the p value of the linear fits.

Is the rationale for developing the new method (or application) clearly explained?

Yes

Is the description of the method technically sound?

Partly

Are the conclusions about the method and its performance adequately supported by the findings presented in the article?

Yes

If any results are presented, are all the source data underlying the results available to ensure full reproducibility?

Yes

Are sufficient details provided to allow replication of the method development and its use by others?

Yes

Reviewer Expertise:

Analytical chemistry, isotopic studies, marine biogeochemistry, biological carbon pump

I confirm that I have read this submission and believe that I have an appropriate level of expertise to confirm that it is of an acceptable scientific standard, however I have significant reservations, as outlined above.

## References

[1] : Estimating precision using duplicate measurements. *J Air Waste Manag Assoc* .2009;59(9) : 10.3155/1047-3289.59.9.1032 1032-9 10.3155/1047-3289.59.9.1032 19785269

# Response

**Published:** 2022-07-20

**Authors:** Giorgio Dall'Olmo

We thank very much Dr Planchon for the time he has taken to review our work and for his thoughtful and constructive criticisms. We have addressed all his comments and have incorporated (where needed) changes in the new version of the manuscript.  

*REVIEWER’S COMMENTS*: *The manuscript presents an interesting and detailed dataset on the occurrence of POC and associated uncertainties in the upper water column of the Atlantic Ocean. This subject is relevant since POC concentrations especially in the mesopelagic zone remain poorly documented at a global scale. In addition, and despite decades of POC measurements, large discrepancies remain between the different methodologies used in terms of sampling techniques, sample preparation, storage, analysis and data validation. The present work, which is a revised version based on the comments of two reviewers, is a further contribution.*

*The main objectives of the work are to quantify the uncertainties related to POC determination based on two independent approaches, the first one with duplicate samples and the second one with a detailed uncertainty budget taking into account three distinct contributions (volume, analytical determination, sample processing). These two estimates of the overall precision of the method are compared and discussed. The most important results of this study are that the determination of POC in the upper water column remains imprecise, especially in the mesopelagic zone (up to 35% error at 68% confidence interval) and adsorption of DOC represents a major source of bias that requires to be considered when POC is obtained from small volume seawater samples. The latter result is not surprising and has already been documented in a number of studies and should be better underlined in the abstract. The former result (precision deduced from duplicate samples) is far less documented and represents a valuable contribution that deserves publication.*

**RESPONSE:** We agree with the reviewer that the uncertainty of POC measurements is not well documented. However, we did not mean to report our results on the adsorption of DOC as a novel result. As the reviewer mentioned, this is a well-known result. What we think is novel about the aDOC section (3.7) is that, because we have a large number of aDOC samples, we could test the impact of i) having these measurements available to correct the uPOC concentrations; ii) having only a small subset of these aDOC samples and using the statistics of these samples to correct the uPOC samples from the entire cruise; or iii) not having them at all. We demonstrated that a good compromise could be to collect a limited number of deep aDOC samples. We believe that this analysis both confirms the importance of collecting aDOC measurements, and also provides a simple solution to minimise filtration times.

**ACTION:** We have modified the original text and added references to section 3.7 to better explain that the biases introduced by the adsorbed DOC are not new. Because the result is not new and we had not mentioned anything about the aDOC biases in the abstract, we did not think it was necessary to add any comment about this to the abstract.

*Regarding the methodology for estimating the precision, the duplicate sample approach is not common and probably suffer from a statistical significance as already mentioned by one reviewer. However, and as mentioned by the authors, this represents a valuable compromise.*

**RESPONSE:** We agree with the reviewer that the method is not common, but given the large volumes of water that needs to be filtered, and as the reviewer recognises, it is a valuable compromise. As we have already discussed in one of our responses to the first round of reviews, statistical significance was achieved by combining the results of all duplicates and by considering their relative differences. In other words, we did not use single duplicate pairs by themselves, but we analysed the statistics of their population. Of course, this technique only allowed us to derive an “across-cruise” estimate of the uncertainty based on the duplicates.

**ACTION:** We added text to section 2.1 to explain why we collected only one duplicate per cast. In the same section, we have also explained that the whole duplicate population is used to derive "overall-cruise estimates of the relative POC experimental uncertainty".

*As a general comment, the paper reads well and is relatively well illustrated. The material and method section describes in much detail the samples considered, the analytical determination and the statistical analysis applied to the data. However, some points need clarification and better organization. Results should not be included in this section as much as possible. For instance, Figure 2 is presented in section 2.4.1 and is not necessary for the total experimental uncertainty calculation since no correlation is assumed between duplicates and the total uncertainty is calculated essentially using the scaled relative difference. It would be more appropriate to present the lack of correlation in the result section to confirm that your calculation is appropriate.*

**RESPONSE:** We respectfully disagree. Figure 2 is necessary to demonstrate a crucial part of our method, namely that we needed to work with relative rather than absolute duplicate differences, because the magnitude of the duplicate differences depended on the POC concentration.

**ACTION:** We have modified the text in section 2.4.1 to make this point more clear.  

*Another occurrence of a result presentation can be found p10 after equation 11.*

**RESPONSE:** We agree.

**ACTION:** We have removed the text after eq 10 presenting the results of the percent uncertainties in POC.  

*Table 2, which summarizes the different contributions, should also be moved to later in manuscript and not introduced in section 2.5.*

**RESPONSE:** We agree.

**ACTION:** We have moved the presentation of the old Table 2 (now Table 3 in the revised version) to the result section.

*Similarly, the section 3.2 dealing with the detection limit should be moved to the method section, at least for the details of the calculation.*

**RESPONSE:** We agree.

**ACTION:** We have now added a new subsection (2.5) to the methods to describe how the detection limit was estimated.

*Regarding the detection limits, you choose to apply a 95% confidence interval, which makes sense and is commonly adopted in analytical chemistry. Why all other uncertainties are at 68% (1 sigma)?*

**RESPONSE:** All other uncertainties are reported at the 1-sigma level to conform to the well-known concept of "standard deviation". It should be easy for a reader to multiply these uncertainties by ~2 to obtain estimates of the 95% confidence intervals.

**ACTION:** None taken.

*Still on the Ld and Lc values and as it can be deduced from Fig 4. It appears that all procedural blanks are below the Ld, only field blanks (aDOC) and samples (uPOC filters) are above. Does it make sense to report a C quantity that is below the Ld? It could even be worse if you considered the quantification limit.*

**RESPONSE:** We understand the reviewer's concern. Yet, we still believe it is useful to report results from all the measurements we have conducted, so that our results can be compared to those from other studies.

**ACTION:** None taken.

*Regarding the total experimental uncertainty (section 2.4.1), I do not understand why it is necessary to apply a propagation law for estimating the precision (Eq. 7, 8, 9). Eq 10 related to the scaled relative difference of duplicates, which is actually your estimator of the relative precision, does not imply such a calculation. Absolute precision can be easily deduced by considering the average POC concentration of each duplicate.*

**RESPONSE:** We thank very much the reviewer for spotting this mistake in our methods. 

**ACTION:** We have now revised sect 2.4.1 and removed the old equations 7-9.

 Accordingly, we have revised all quantitative results and figure related to the POC uncertainty. This mistake resulted in just small changes in our quantitative results and did not change our results qualitatively.  

*Still on this parameter (σΔr) and as mentioned in Hyslop and White (2009), it could be worth checking that your scaled relative differences are normally distributed. In that studies, three different estimators of the precision have been used including the root mean square (RSM), the scaled mean absolute difference (MAD) and the percentiles approach. Why did you choose the percentile precision?*

**RESPONSE:** It is not a requirement that the scaled relative differences are normally distributed. If they are, the different metrics proposed in Hyslop and White 2009 will deliver approximately equivalent results. In our case, the distributions of the scaled relative differences are affected by outliers. The influence of these outliers on our metrics needs to be minimised, because we want to derive an estimate of the uncertainty for all measurements collected during the cruise. The percentile approach is the least sensitive to outliers (Hyslop and White, 2009). This is why we have selected the percentile range as a metrics (eq. 7 in the revised text).

**ACTION:** To explain the above argument in the manuscript, we have modified the text related to equation 7 in the revised text.

*Another issue concerns the section 2.5.2 (uncertainty in mass predicted by the calibration equation), which is essentially the instrumental standard deviation. I do not understand why you include σMp in this section, which refers to the correction of total POC mass with the corresponding DOC mass. This parameter is not related to the calibration equation and represent a post-analysis correction made possible with your sampling methodology. I think it could be interesting to consider separately these two components (instrument precision and precision on the corrected POC quantity by the DOC quantity). At this point it is not clear what the values reported in table 2 and associated with the calibration correspond to, instrument precision or precision on the corrected mass.*

**RESPONSE:** Our objective in this section was to propagate the uncertainty of the calibration equation to the POC concentration. We agree with the reviewer that the original text was not clear enough. But we have already dedicated one section to the impact of how the aDOC correction can affect the resulting POC, therefore we have decided to focus this section on the impact of the uncertainties in the calibration equation to the final POC concentration.

**ACTION:** We have modified the text in this section ("Uncertainty in POC concentration due to uncertainties in the calibration equation ") to clarify that we want to estimate the impact of the calibration equation on the POC concentration. We have also clarified this in Tables 3 and 4 of the revised manuscript.

*Still on the uncertainty budget in the section 2.5.3 (uncertainty due to sample handling during CHN analysis), the proposed calculation based on the three acidified filter blanks does not only correspond to contamination due to the acidification step but includes also the contribution from the filter. In Eq 17, I do not think it is necessary to introduce two distinct variables (ση1 and 2) since the two are equal and are estimated from the three acidified filter blanks. It could be interesting to consider separately these two distinct sources of uncertainty. Impact of acid fuming by considering the difference between non-acidified and acidified filter and the filter alone.*

**RESPONSE:** We agree with the reviewer: the variability of the carbon mass of the acidified filters depends both on the uncertainties in measuring carbon on the acidified and on the clean filters. However, given that this source of uncertainty contributes a very small fraction of the final uncertainty, we have decided not to separate these two sources of uncertainty.

**ACTION:** We have now modified this section ("Uncertainty due to laboratory contamination") to clarify that it deals with the uncertainty due to filter contamination during laboratory analyses.

 Another remark on the impact of filter concerns their role in the analytical determination and *especially on the sensitivity of the instrument. This is not taken into account in your dataset since your CHN standards were prepared without filters. Filters can affect the combustion process and as a consequence the sensitivity of the instrument (the slope of the calibration curve). Accordingly, it is highly recommended to tune the elemental analyzer with standards containing filters. In general, an increase in O2 gas is necessary to prevent partial combustion of standards and samples. Also, adding a filter to the standards allows for better reproduction of the sample matrix and provides a means for quantifying filter blanks through the regression analysis (b parameter). This procedure is required in C isotope studies for both concentration and isotopic corrections when samples are loaded on a filter.*

**RESPONSE:** We thank the reviewer for this interesting remark. We were not aware of this methodological aspect, nor does it appear in the most updated protocols for POC determination. However, we have thoroughly searched the literature and have not managed to find a reference that describes this methodological detail.

**ACTION:** We have added a sentence to the method section to mention this additional aspect and cited a “personal communication” by Dr Planchon.  “Finally, we note that, in carbon-isotope analyses filters are expected to affect the combustion process and thus the sensitivity of the analyser (Planchon, personal communication, 2022). As a consequence, to reduce uncertainties and improve sensitivity, future studies should consider investigating this effect on POC determinations.”

*The result and discussion section is clearly written with abundant references to previous studies. I have only few concerns on this section. The first one is related to the description of the results. You present the median of your results with an associated uncertainty. Could you clarify how these uncertainties have been obtained (number of values and calculation)? My guess is that it corresponds to the standard deviation of your data.*

**RESPONSE:** The reported uncertainties are robust standard deviations computed as described by eq. 9 (after substituting \Delta_r with the appropriate variable for which the uncertainty is being estimated). The number of samples for the productive and mesopelagic zones are reported in Table 2 of the revised manuscript.

**ACTION:** In the revised manuscript, we have added a new subsection (2.6) to the methods to explain how uncertainties associated with medians were computed. We have also mentioned at the start of the Results section that the number of samples for each zone is reported in Table 2.  

*Looking at these uncertainties, they appear to be highly variable indicating a high dispersion of uncertainty data. I think this dispersion could be better considered and at least mentioned, especially when you compare the productive zone with the mesopelagic zone. For instance in section 3.7 when you compare uPOC with POC concentrations, I do not think that the difference between sampling zones is statistically different.*

**RESPONSE:** Our intention in Sect 3.7 was not to state that the comparison between uPOC and POC was different between the productive and mesopelagic zones. What we wanted to do was to show that uPOC was higher than POC and therefore that the aDOC blank subtraction was important (as already well known). We presented separate results for productive and mesopelagic zones because throughout the manuscript the two zones are always described separately. In general, the uncertainties associated with the median values reported represent the spatial variability of the dataset in the two zones.

**ACTION:** In the revised manuscript (sect 3.1), we have added a sentence to clarify that these uncertainties represent the spatial variability of the dataset.

*Another point concerns the observed positive correlation between aDOC and POC concentrations (section 3.3 and figure 9). In addition to cells break and release of DOC, another hypothesis may concern the potential impact of small (<0.7 µm) particles associated with picophytoplankton (0.2-2µm in size) and small heterotrophs (free bacteria, archea, etc.). These organisms can be abundant in surface waters representing an important part of the biomass especially out of the bloom period.*

**RESPONSE:** We agree with the reviewer although these particles smaller than 0.7um, if important, should also pass through the aDOC filter. We agree however that having two stacked filters increases the chances that a small particle is retained.

**ACTION:** In the revised manuscript we have added text (just above the beginning of section 3.4) to include the reviewer's suggestion as a potential alternative explanation of the aDOC-POC correlation.

*Detailed comments:*

*Abstract L6-7: check sentences and change accuracy term with precision.*

**RESPONSE:** We agree.

**ACTION:** Done.

*Section 2.1: clarify the number of samples taken at each station, were they taken at the same cast? You mention 392 uPOC samples. if you collected 6 samples at 67 stations, this makes a total of 402 samples.*

**RESPONSE:** We agree that the numbers did not added up correctly, because we had not mentioned exceptions (e.g., lost samples).

**ACTION:** We have now added information that should allow the reader to verify the number of samples (sect 2.1).

*Similarly, you mention duplicate samples were taken at each cast. You should have 67 duplicate samples and not 57. Please clarify*

**RESPONSE**: We agree that the numbers did not added up correctly, because we had not mentioned exceptions (e.g., lost samples).

**ACTION:** We have now added information (sect 2.1) that should allow the reader to verify the number of duplicates.

*Page 5, last paragraph: if filter blanks were placed in the dessicator they are obviously acidified. I would change to: process as filter sample and subjected to acid fuming in the dessicator.*

**RESPONSE:** We agree.

**ACTION:** Done.

*Page 6, third paragraph: correct your description of the sample analysis in the CHN. The samples are combusted in the oven (combustion chamber subjected to a dedicated temperature program), and then ashes fall in the ash trap, which progressively fills up. This is not a reaction tube. Then combustion gases are carried to the oxidation and reduction “tubes” (chambers) and then to the chromatographic column for separation and final detection.*

**RESPONSE:** Thank you.

**ACTION:** We have now corrected the sentence.

*P6, paragraph 4-5: Theoretically, all standards measured require to be included in the calibration. The first set called “calibration standards” are generally used to check the linearity. For state of the art analytical sequence, linearity is checked also at the end of the sequence. Also, standards showing poor recovery (due to weighting error, bad analytical run) can be removed according to z-score.*

**RESPONSE:** We thank the reviewer for this clarification. At the time of the analysis we were not aware that linearity should be also checked at the end of the analytical sequence. The calibration sequence we used was defined based on the manufacturer recommendation. Furthermore, even the most recent protocols for POC determination do not specify this is a requirement. Nevertheless, we agree with the reviewer about this potential additional check that can be performed on the analytical sequence. We also thank the reviewer for the suggestion to use the z-score metrics to flag outliers in the calibration process. As discussed in the text, the instrument instability was detected as an increased noise (but not a bias) in most of the stability standards, rather than in few of them. That is why we decided to not remove outliers, but to use all standards to generate a calibration equation.

**ACTION:** In the revised text (4th paragraph in sect 2.3) we have added the following sentence: "To better characterise the instrument's behaviour during the analysis, future work should also use standards of with different weights at the end of the CHN run."

*P6, paragraph 6: you often use the term robust throughout the manuscript (here for the fitting model), what does it mean? Does it differ from a classical linear fitting model?*

**RESPONSE:** Yes it does. This is a fitting algorithm ("iteratively reweighted least-squares") that reduces the influence of outliers on the estimate regression parameters (i.e., slope and intercept)

**ACTION:** In the revised text we have clarified this in the paragraph before eq 1.

*P6, bottom page: the two equations are not numbered and the definition of M star is not given. Furthermore, there is a missing term in these two equations corresponding to the C mass associated to the filter. The difference between acidified and non-acidified filter blanks corresponds only to the C added by the acidification process.*

**RESPONSE:** We agree.

**ACTION:** Corrected.

*P8, last paragraph: indicate the p value of the linear fits to explore the significance of the regression analysis.*

**RESPONSE:** We agree.

**ACTION:** Done.

*P9, figure 2: y axis of figure 2b is unitless.*

**RESPONSE:** Thanks.

**ACTION:** Corrected.

*P10, section 2.5: an equation showing how you compute all uncertainty contributions would help the reader.*

**RESPONSE:** We agree.

**ACTION:** In the revised text we have now added a new equation (11) to describe how the uncertainty contributions were computed.

*P10, Table 2: to be clearer, add a column with the corresponding variables associated with each line. If this table is moved to the result section, please include the sd for each median value and adjust to significant digit.*

**RESPONSE:** We agree.­

**ACTION:** Done.

*P14, last paragraph: indicate the p value of the linear fits and homogenize the significant digit.*

**RESPONSE:** We agree.

**ACTION:** Done.

*P17, table 4: same remarks as for table 2.*

**RESPONSE:** We agree.

**ACTION:** Done.

*P18, Figure 9: indicate the p value of the linear fits.*

**RESPONSE:** We agree.

**ACTION:** Done.

# Peer review: Reviewer response for version 1

**DOI:** 10.21956/openreseurope.14465.r26795

**Published:** 2021-05-17

**Authors:** Anna Belcher

**Affiliations:** 1 Ecosystems Team, British Antarctic Survey, Cambridge, UK

The manuscript aims to develop a method to quantify the experimental uncertainties of POC concentrations, and hopes to contribute to developing a uniform protocol. Quantifying POC in the ocean is important and thus ensuring that a reliable and standardised method is adopted amongst researchers is important. Ensuring appropriate blanks are taken and that the necessary corrections are made is key, and the corrections made need to be transparent in each study, and ideally consistent amongst the research community.

 The manuscript is clearly written and the authors have presented a clear explanation of the uncertainties that they have investigated. It is good to see each stage of the sampling to measurement process investigated, however there are some gaps in this which limit the uncertainty assessment. Particularly, certified reference materials should have been used to assess the accuracy of the CHN analysis. Additionally, it is a shame that a filtration blank was not taken – as the authors mention themselves, using Mili-Q water and an additional blank.

 It is good to see the consideration of filtration time when assessing the use of double stacked filters, as this may well be a factor for many studies. Although the level of acceptable uncertainty may be different for different studies, it would be good for a ‘gold standard’ method to be identified by the authors in terms of giving their recommendation.

 The distinction between DOC and POC is an interesting one, particularly when thinking about DOC contained within particles. A second filter is used in the study to estimate the concentration of DOC that is adsorbed onto the filter, which can be used to correct for DOC. However, as stated in section 3.3, fragmentation of particles into smaller particles could contaminate the aDOC filters. Additionally, particles will also contain DOC trapped within the particle matrix, some of which may be released during filtration due to damage of particles and DOC leakage. The loss due to DOC leakage with the break up of particles on the filter is difficult to quantify, but poses an interesting question as to what we want to measure when we take a sample for POC. Gaining a measure of the ‘true’ POC in the water column is useful – i.e. correcting for DOC contamination, but if we want to think about the total carbon that can be transported by particles, then we should consider the trapped DOC in the particles. In a detailed uncertainty assessment such as this study, it would be worth mentioning/discussing this as a point to consider when we make POC measurements.

 Although, the authors provide a useful assessment of uncertainty with clear and careful calculations, there are a few aspects of the work that make the uncertainty assessment incomplete. There is merit in the work undertaken and useful information for the scientific community, but some of these missing aspects need to be acknowledged and discussed further, and the limitations of the presented uncertainty values made clear. The manuscript therefore needs revisions before it can be considered to be indexed.

**Specific comments:**

 Introduction, second paragraph, end of first sentence, ‘coverage of POC’, perhaps clearer to add ‘POC estimates’? If this is what you mean?

 It would be useful in the introduction to give more information on the IOCCG and JGOFS protocols in terms of what is missing from these, and how this paper will address these.

 Section 2.3 – The authors say that constant adjustments to the CHN analyser were made. What are these, if these is important for accuracy then needs to be stated for reproducibility.

 Section 3.3 – if aDOC and POC concentrations are spatially correlated as the authors suggest, it would be good to see the aDOC and corresponding POC value plotted against one another. Figure 5 and figure 3 are hard to compare for this purpose. It would be good to do this for mesopelagic and surface values separately, as this would also allow to separate out any depth driven correlation.

 Section 3.7 – Please make clearer what you mean by ‘original POC concentrations’ as this is vague.

 Not all variables are defined in table 1.

 Table 3: Add units.

 Figure 4: should be ‘black’ not ‘blank’.

Is the rationale for developing the new method (or application) clearly explained?

Partly

Is the description of the method technically sound?

Partly

Are the conclusions about the method and its performance adequately supported by the findings presented in the article?

Partly

If any results are presented, are all the source data underlying the results available to ensure full reproducibility?

Partly

Are sufficient details provided to allow replication of the method development and its use by others?

Partly

Reviewer Expertise:

Biological carbon pump, ocean biogeochemistry.

I confirm that I have read this submission and believe that I have an appropriate level of expertise to confirm that it is of an acceptable scientific standard, however I have significant reservations, as outlined above.

# Response

**Published:** 2021-08-27

**Authors:** Giorgio Dall'Olmo

**Reviewer comment: **General comments. The manuscript aims to develop a method to quantify the experimental uncertainties of POC concentrations, and hopes to contribute to developing a uniform protocol. Quantifying POC in the ocean is important and thus ensuring that a reliable and standardised method is adopted amongst researchers is important. Ensuring appropriate blanks are taken and that the necessary corrections are made is key, and the corrections made need to be transparent in each study, and ideally consistent amongst the research community. The manuscript is clearly written and the authors have presented a clear explanation of the uncertainties that they have investigated. It is good to see each stage of the sampling to measurement process investigated, however there are some gaps in this which limit the uncertainty assessment. Particularly, certified reference materials should have been used to assess the accuracy of the CHN analysis. Additionally, it is a shame that a filtration blank was not taken – as the authors mention themselves, using Mili-Q water and an additional blank.

**Response: **We thank very much the reviewer for the time they have spent in commenting our manuscript. We agree that a certified reference material (CRM) is needed for POC measurements, but as of now, no such CRM has been agreed by the community (see also responses to reviewer 1). We also agree that adding procedural filters would be helpful and that is why we have for example introduced blank filters to quantify the contamination that could occur during the acidification step. However, before calculating the uncertainty budget, we were not aware of the potential *critical *role of sources of uncertainties different from the CHN calibration and the DOC adsorption on filters. As such, we had not planned for testing contamination from, for example, the atmosphere of the ship’s laboratory. Our hope is that by presenting our uncertainty budget and the disagreement between experimental and modelled uncertainties and by listing potential sources of uncertainty, in future studies such uncertainties will be addressed, quantified, and minimised.

**Action:** None taken. 

**Reviewer comment: **It is good to see the consideration of filtration time when assessing the use of double stacked filters, as this may well be a factor for many studies. Although the level of acceptable uncertainty may be different for different studies, it would be good for a ‘gold standard’ method to be identified by the authors in terms of giving their recommendation.

**Response: **We do not think it is up to us to define a “gold standard” for the community to follow. If at all, the definition of “gold standard” should be a community effort. We believe the standards to follow depend on the level of accuracy that is required to answer a specific question using data from a specific ocean region. What we have done is to quantify the additional uncertainties introduced by not collecting an aDOC sample for every uPOC sample (section 3.7). We expect these findings to guide future studies in potentially reducing filtration times.

**Action:** None taken.  

**Reviewer comment: **The distinction between DOC and POC is an interesting one, particularly when thinking about DOC contained within particles. A second filter is used in the study to estimate the concentration of DOC that is adsorbed onto the filter, which can be used to correct for DOC. However, as stated in section 3.3, fragmentation of particles into smaller particles could contaminate the aDOC filters. Additionally, particles will also contain DOC trapped within the particle matrix, some of which may be released during filtration due to damage of particles and DOC leakage. The loss due to DOC leakage with the break up of particles on the filter is difficult to quantify, but poses an interesting question as to what we want to measure when we take a sample for POC. Gaining a measure of the ‘true’ POC in the water column is useful – i.e. correcting for DOC contamination, but if we want to think about the total carbon that can be transported by particles, then we should consider the trapped DOC in the particles. In a detailed uncertainty assessment such as this study, it would be worth mentioning/discussing this as a point to consider when we make POC measurements.

**Response: **We agree that particles (e.g., cells) contain DOC. Yet, this DOC contained in particles is operationally defined as part of the POC (unless particle breaks and this DOC is released). The concentration of this particle-related DOC is expected to be significantly smaller than the ambient DOC. To determine the extent to which this particle-related DOC should be a major avenue of future research, dedicated experiments should be devised and conducted to quantify how much DOC is contained in cells.

**Action:** We added a sentence to make the reader aware that broken cells release their inner DOC in the environment.  

**Reviewer comment: **Although, the authors provide a useful assessment of uncertainty with clear and careful calculations, there are a few aspects of the work that make the uncertainty assessment incomplete. There is merit in the work undertaken and useful information for the scientific community, but some of these missing aspects need to be acknowledged and discussed further, and the limitations of the presented uncertainty values made clear. The manuscript therefore needs revisions before it can be considered to be indexed.

**Response: **We thank again the reviewer for their suggestions.

**Action:** We have addressed all questions/comments (see below).  

**Reviewer comment: **Specific comments. Introduction, second paragraph, end of first sentence, ‘coverage of POC’, perhaps clearer to add ‘POC estimates’? If this is what you mean? It would be useful in the introduction to give more information on the IOCCG and JGOFS protocols in terms of what is missing from these, and how this paper will address these.

**Response: **We agree.

**Action:** We have replaced “coverage” with “measurements”. We have added a paragraph at the beginning of Section 2 in which we introduce the two protocols. However, since we have also focused the manuscript more on quantifying the POC uncertainties and their sources, we have not presented a detailed description of these protocols.  

**Reviewer comment: **Section 2.3 – The authors say that constant adjustments to the CHN analyser were made. What are these, if these is important for accuracy then needs to be stated for reproducibility.

**Response: **This was clarified just after the words “constant adjustments”, i.e., “to sample delay, which represents the time that it takes the CHN analyser to combust each sample, to deliver CO2 released from the sample to the detector, and to run the analysis.”

**Action:** We have now written “sample delay” in quotes so that the reader is aware that the next part of the sentence explains what the adjustments were referring to. We hope this help making the text clearer.  

**Reviewer comment: **Section 3.3 – if aDOC and POC concentrations are spatially correlated as the authors suggest, it would be good to see the aDOC and corresponding POC value plotted against one another. Figure 5 and figure 3 are hard to compare for this purpose. It would be good to do this for mesopelagic and surface values separately, as this would also allow to separate out any depth driven correlation.

**Response: **We agree.

**Action:** We have now added a new figure plotting aDOC vs POC and added related text with the correlation coefficients for the mesopelagic and productive zones.  

**Reviewer comment: **Section 3.7 – Please make clearer what you mean by ‘original POC concentrations’ as this is vague.

**Response: **Thank you for pointing out this issue.

**Action:** We have now removed “original”, which should make the text clearer.  

**Reviewer comment: **Not all variables are defined in table 1. Table 1 is a non-exhaustive list, please include the rest of variable.  Table 3: Add units.

**Response: **Thank you for pointing out these issues.

**Action:** We have now updated the Tables 1 and 3.  

**Reviewer comment: **Figure 4: should be ‘black’ not ‘blank’.

**Response: **Thank you for pointing this out.

**Action:** Corrected.

# Peer review: Reviewer response for version 1

**DOI:** 10.21956/openreseurope.14465.r26796

**Published:** 2021-05-10

**Authors:** Eric Achterberg

**Affiliations:** 1 Marine Biogeochemistry Division, GEOMAR Helmholtz Centre for Ocean Research, Kiel, Germany

The submitted manuscript deals with an uncertainty assessment for the measurement of POC in oceanic water columns. POC is relevant as its removal through sinking from the surface ocean provides a means of long-term sequestration of carbon in the deep ocean.

 POC levels are low in the ocean, and its measurement is influences by a range of factors which effect data quality. A range of papers have been published on this issue, and the submitted manuscript aims to make a further contribution.

 The paper makes a nice assessment of the various uncertainties associated with oceanic POC measurements, and attempts to quantify the uncertainties. For a range of uncertainties, it was not possible to constrain them, as the experimental set-up was inappropriate.

 The starting point for the work was duplicate sampling and analysis of a subset of POC samples on a research cruise. The difference between the duplicate samples formed the basis of the work in the manuscript. It seems that the overall uncertainty work was not the result of a carefully planned experiment prior to the cruise, but an afterthought following the cruise. Unfortunately this shows in the manuscript, and seriously impacts quality.

 In case a thorough uncertainty assessment was to be planned, then instead of duplicate samples, the authors would have sampled 5 samples, allowing thorough statistical assessment. The sampling equipment, and sample processing equipment (incl containers) would have been assessed for contamination risk/level. Filtration instrument blanks would have been collected (with and without clean MQ water filtration), and also filtering would have been conducted under more controlled conditions on-board ship.

 The analytical analysis is lacking a certified reference material measurements, which hampers accuracy assessment.

 Overall, the manuscript provides a detailed assessment of various causes for uncertainty, but the unfortunate experimental set up precludes meaningful conclusions to be drawn from the work. A dedicated experiment will need to be conducted to assess POC measurement uncertainty, with careful planning prior to the experiment which will allow for sound statistical data analysis. I cannot support indexing of this manuscript.

**Specific comments**

 Intro: first line. Please be very careful in the distinction between POC and OM. POM is composed of POC, POC is not composed of POM. Rephrase sentence.

 …..extensive coverage of POC? Coverage of what aspect of POC? Explain.

 …methods for determining POC? Unclear what you are referring to. The CHN analysis is well established. Which methods are you referring to? I assume it is the sampling, and sample processing and filter blank assessment rather than the laboratory analysis?

 Accuracy assessment for POC. This can be achieved for the laboratory analysis using certified reference materials, but not for the overall process of sampling, sampling processing and lab analysis. Intercomparisons etc. can be conducted for data validation purposes.

 The intro would benefit from a better context of the topic. In 2 sentences, place POC in the context of DIC and DOC, and PIC. POC is of course the C fraction occurring at the lowest concentration.

 The authors should also mention the particle sizes that they are referring to when discussing POC (rather than DOC).

 Data and methods.

 I suggest that the authors only use the term accuracy when a referring to the analysis of a certified reference material.  

 Section 2.1 Provide clear details on sampling approach. Including make of bottles and type of rosette frame.

 Analysis of duplicate samples does not provide information on accuracy. It provides information on replication.

 Section 2.2. Were procedural filters analysed (filters placed in filtration set-up at sea, with no seawater passed over them)?

 Why is it important to reduce humidity in the desiccator.

 Provide details on acetanilide.

 Page 6…Therefore, the true mass… Please avoid the term true, as you cannot prove this. You mean ‘blank corrected’, I assume.

 2.5.2. It is worrying that there is a weighing error introduced into the overall method assessment. This undermines all the conducted work.

 There are certified reference materials (soil material), which can be analysed as part of the calibration, and also a regular check sample during the analyses. This will provide the researcher with more confidence in the measurement quality, which currently is unconstrained. Hence, the word accuracy and accurate should not appear in the manuscript.

 Results & discussion. I do not understand why the authors say the unit mg/m ^3^. I think using a unit with carbon in moles, and then L (or m ^3^) or kg, would be more appropriate.

 Fig. 3 Colour differences between samples is hard to decipher.

 P 15, bottom sentence: this statement does not allow for a comparison with the quantitative uncertainty approach conducted by the authors. Please quantify the potential consequence of the large particles.

Is the rationale for developing the new method (or application) clearly explained?

Partly

Is the description of the method technically sound?

Partly

Are the conclusions about the method and its performance adequately supported by the findings presented in the article?

Partly

If any results are presented, are all the source data underlying the results available to ensure full reproducibility?

Partly

Are sufficient details provided to allow replication of the method development and its use by others?

No

Reviewer Expertise:

Chemical oceanography, marine chemistry, carbon export, carbon cycle.

I confirm that I have read this submission and believe that I have an appropriate level of expertise to state that I do not consider it to be of an acceptable scientific standard, for reasons outlined above.

# Response

**Published:** 2021-08-27

**Authors:** Giorgio Dall'Olmo

**Reviewer comment: **General comments: The submitted manuscript deals with an uncertainty assessment for the measurement of POC in oceanic water columns. POC is relevant as its removal through sinking from the surface ocean provides a means of long-term sequestration of carbon in the deep ocean. POC levels are low in the ocean, and its measurement is influences by a range of factors which effect data quality. A range of papers have been published on this issue, and the submitted manuscript aims to make a further contribution.

**Response: **We thank the reviewer for the time they invested in providing comments to our draft manuscript. These comments have allowed us to improve the presentation of our work in the revised version. 

**Reviewer comment: **The paper makes a nice assessment of the various uncertainties associated with oceanic POC measurements, and attempts to quantify the uncertainties. For a range of uncertainties, it was not possible to constrain them, as the experimental set-up was inappropriate. The starting point for the work was duplicate sampling and analysis of a subset of POC samples on a research cruise. The difference between the duplicate samples formed the basis of the work in the manuscript. It seems that the overall uncertainty work was not the result of a carefully planned experiment prior to the cruise, but an afterthought following the cruise. Unfortunately this shows in the manuscript, and seriously impacts quality. In case a thorough uncertainty assessment was to be planned, then instead of duplicate samples, the authors would have sampled 5 samples, allowing thorough statistical assessment. The sampling equipment, and sample processing equipment (incl containers) would have been assessed for contamination risk/level. Filtration instrument blanks would have been collected (with and without clean MQ water filtration), and also filtering would have been conducted under more controlled conditions on-board ship. 

**Response: **The “uncertainty work” we have presented in the manuscript can be divided into two separate sections. First, we have presented an empirical method to estimate the uncertainty of POC estimates based on duplicate samples: to the best of our knowledge the technique we presented has not been used for POC before, so we believe it is worth publishing on its own. Second, we have modelled the uncertainty of our POC measurements, based on a limited set of input sources of uncertainty. Finally, by comparing the empirical and modelled uncertainties, we showed that the latter accounted for a relatively small fraction of the empirical uncertainties, strongly suggesting that we did not include some important sources of uncertainties in our modelled uncertainty. The first part of our uncertainty work, was planned before the cruise. That is why we collected duplicate measurements at all stations, as well as aDOC blanks for each sample. The reviewer states that, had we planned this work in advance, we would have collected five (it is not clear why exactly five) replicate samples, rather than the duplicates we collected. We agree that larger number of replicates could have improved our estimates of the uncertainties, for example by allowing us to determine an uncertainty for each specific measurement. This reviewer, however, does not seem to be taking into consideration the large water volumes that we did sample (up to 8 litres, another pre-cruise planned activity to minimise uncertainties in the low-signal waters of the mesopelagic) and the constraints imposed on how much water was available from each cast for each scientist during the field campaign. Put simply, there would not have been enough water for the rest of the science party if we had collected five replicates at each station. Instead, we opted for a practical compromise and collected duplicates at different depths and used the statistics of the relative differences between the duplicates to quantify the overall uncertainty of our POC measurements during the entire field campaign. These duplicate uncertainties are very useful in putting all the measured values into context. The second part of our “uncertainty work”, the uncertainty budget, instead was an exercise that we decided to undertake after the cruise to better understand how POC measurements can be improved. We agree that this exercise has not provided definitive answers, but it has nevertheless clearly indicated that the sources of uncertainty we initially assumed to be more important (i.e., the calibration of the CHN analyser, aDOC correction) were NOT the dominant sources of uncertainty in the measurements. We have therefore discussed several potential sources of uncertainty that should be investigated in the future. Despite the limitations of this section, we still believe it is worth publishing as it can change attitudes on the dominant sources of uncertainty and therefore guide future studies. Finally, we would like to note that in 2014 (when we collected the samples described in this manuscript) the POC protocols (Knapp *et al.*, 1996) did not describe all the potential sources of uncertainties affecting POC measurements that we have presented in our manuscript. As a consequence, our thinking was guided by previous POC measurements we had collected and on the literature we were familiar with. Only recently has a new version of the POC protocol appeared (still in draft form) and we are part of this new effort to improve the quality of POC determinations ( https://ioccg.org/wp-content/uploads/2019/11/poc_ioccg_protocol_2019_public_draft-18nov-2019.pdf). In other words, had we known all the major potential sources of uncertainty, we would have collected additional measurements to quantity their contributions to the total uncertainty. To conclude, while analytical protocols for other chemical species (e.g., macro- and micro-nutrients) may be well established, the situation is different for POC. Clear protocols are not yet well established, intercomparisons between laboratories are not routinely conducted, and there is no certified reference material for POC. As a consequence, POC measurements are still relatively uncertain, especially in the mesopelagic, where POC is extremely low.

**Action: **We clarified in the introduction the two different parts of the “uncertainty work”. We also improved the method section explaining what protocols were available at the time of sampling and what has appeared later.

**Reviewer comment: **The analytical analysis is lacking a certified reference material measurements, which hampers accuracy assessment.

**Response: **We agree with the reviewer that a certified reference material (CRM) would be desirable to assess the accuracy of our measurements. However, as of today, no such reference material is available. As the reviewer suggest, there exist some CRMs that might be used, but these are all based on sediment samples, with very different matrices from open-ocean pelagic particulate matter (National Research Council. "Chemical Reference Materials: Setting the Standards for Ocean Science". The National Academies Press, Washington, DC, 2002.  ISBN 978-0-309-08500-7. doi: 10.17226/10476. https://www.nap.edu/catalog/10476/chemical-reference-materials-setting-the-standards-for-ocean-science.) In addition, not all bio-organic elements of these CRMs have been quantified, making it difficult to assess how similar they are to open-ocean pelagic sediments. Thus, these existing materials are not recognised by the POC community as reference materials for POC and are not routinely used to assess the accuracy of POC measurements ( https://ioccg.org/wp-content/uploads/2019/11/poc_ioccg_protocol_2019_public_draft-18nov-2019.pdf).

**Action:** In the revised version of the manuscript, we have added a new section discussing the need for CRMs for POC analyses. 

**Reviewer comment: **Overall, the manuscript provides a detailed assessment of various causes for uncertainty, but the unfortunate experimental set up precludes meaningful conclusions to be drawn from the work. A dedicated experiment will need to be conducted to assess POC measurement uncertainty, with careful planning prior to the experiment which will allow for sound statistical data analysis. I cannot support indexing of this manuscript.

**Response: **We agree with the reviewer that more work is needed to identify all sources of uncertainty for POC and we have listed several potential ones in our manuscript. However, we disagree that the work we have conducted and described in this manuscript is not worth publishing. First, we have presented a dataset of POC measurements in the mesopelagic (0-500m) along an Atlantic Meridional Transect. By itself such a dataset is very useful, for example to assess model output. Second, we have presented a detailed method to quantify experimentally the total uncertainty associated with these POC measurements based on duplicate samples. This is important because it will allow other researchers to similarly quantify and report their uncertainties, which is key to assess and compare datasets, especially in the mesopelagic where signals are very low. Third, we have modelled the total POC uncertainty based on what, at the time of the analysis, were thought to be the largest sources to the uncertainty in POC. Results from this modelling analysis demonstrated that the assumed sources of uncertainty were able to explain only a fraction of the total experimental uncertainty. We have thus listed potential additional sources of uncertainty that should be minimised and quantified in the future. Unfortunately, the reviewer only focused on this last point and did not seem to consider the other parts of our work.

**Action: **We have revised the manuscript to clarify the different contributions of our work. 

**Reviewer comment: **First line. Please be very careful in the distinction between POC and OM. POM is composed of POC, POC is not composed of POM. Rephrase sentence.

**Response: **We agree with the reviewer.

**Action:** We have removed the term “POM” from the manuscript. 

**Reviewer comment: **….extensive coverage of POC? Coverage of what aspect of POC? Explain.

**Response: **We agree with the reviewer.

**Action:** We have replaced “coverage” with “measurements”. 

**Reviewer comment: **…methods for determining POC? Unclear what you are referring to. The CHN analysis is well established. Which methods are you referring to? I assume it is the sampling, and sample processing and filter blank assessment rather than the laboratory analysis?

**Response:** Here we are referring to all the steps needed to determine POC, including sampling, sample processing and acidification, filter blank assessment, as well as CHN analyses. While the CHN analyses are well established, we still do not know how much of the total POC uncertainty they contribute.

**Action:** We have clarified this in the revised version of the manuscript. 

**Reviewer comment: **Accuracy assessment for POC. This can be achieved for the laboratory analysis using certified reference materials, but not for the overall process of sampling, sampling processing and lab analysis. Intercomparisons etc. can be conducted for data validation purposes.

**Response: **No CRMs are available for POC. We agree that intercomparison exercises are needed to quantify and minimise uncertainties due to sample collection and processing before the CHN analysis in the laboratory.

**Action:** We have added a paragraph that specifically discusses the above. 

**Reviewer comment: **The intro would benefit from a better context of the topic. In 2 sentences, place POC in the context of DIC and DOC, and PIC. POC is of course the C fraction occurring at the lowest concentration. The authors should also mention the particle sizes that they are referring to when discussing POC (rather than DOC).

**Response: **We agree.

**Action:** We have now added a part of the introduction that defines POC and places it in the context of the other carbon pools. 

**Reviewer comment: **I suggest that the authors only use the term accuracy when a referring to the analysis of a certified reference material. 

**Response: **We agree.

**Action:** Where relevant, we have replaced “accuracy” with “uncertainty”. 

**Reviewer comment: **Section 2.1 Provide clear details on sampling approach. Including make of bottles and type of rosette frame.

**Response: **We agree.

**Action:** We have added more details including make of bottles and type of rosette frame. 

**Reviewer comment: **Analysis of duplicate samples does not provide information on accuracy. It provides information on replication.

**Response: **We agree.

**Action:** We have replaced “accuracy” with “uncertainty”, where relevant. 

**Reviewer comment: **Section 2.2. Were procedural filters analysed (filters placed in filtration set-up at sea, with no seawater passed over them)?

**Response: **No procedural filters were used. This is something that we realised is needed, when we computed the uncertainty budget. No such procedural filters were recommended in the Knaps 1996 POC protocols.

**Action:** None taken. 

**Reviewer comment: **Why is it important to reduce humidity in the desiccator.

**Response: **We have some experimental evidence that silica gel in the desiccator reduces the contamination during the acidification step. However, we do not fully understand what processes controls this. This is why we also introduced additional blanks to quantify the contamination.

**Action:** We have removed this sentence. 

**Reviewer comment: **Provide details on acetanilide.

**Response: **Agreed.

**Action: **We have added the manufacturer and model number of the acetanilide used. 

**Reviewer comment: **Page 6…Therefore, the true mass… Please avoid the term true, as you cannot prove this. You mean ‘blank corrected’, I assume.

**Response: **Agreed.

**Action:** Done. 

**Reviewer comment: **Section 2.5.2. It is worrying that there is a weighing error introduced into the overall method assessment. This undermines all the conducted work.

**Response: **We disagree with the reviewer. As stated in the manuscript, “[...] there was no bias in the less accurate standards - they resulted in unbiased calibration coefficients, even though the random uncertainties around these coefficients were higher.” We also disagree that having less accurate, but unbiased, standards in two out of 16 CHN runs “undermines all the conducted work”.

**Action: **None taken. 

**Reviewer comment: **There are certified reference materials (soil material), which can be analysed as part of the calibration, and also a regular check sample during the analyses. This will provide the researcher with more confidence in the measurement quality, which currently is unconstrained. Hence, the word accuracy and accurate should not appear in the manuscript.

**Response: **As stated in a previous response, there are no community agreed certified reference materials for POC. Therefore, the accuracy of POC measurements cannot be quantified at present.

**Action:** Where relevant, we have removed the words “accuracy” and “accurate” from the revised version.

**Reviewer comment: **Results & discussion. I do not understand why the authors say the unit mg/m3. I think using a unit with carbon in moles, and then L (or m3) or kg, would be more appropriate.

**Response: **We disagree. The community dedicated to measuring POC concentrations has not selected official units for POC (e.g., see IOCCG POC protocol draft: https://ioccg.org/wp-content/uploads/2019/11/poc_ioccg_protocol_2019_public_draft-18nov-2019.pdf). Thus, both grams and moles are commonly used for POC. It is easy to convert from POC in grams/m3 to POC mol/m3 by dividing the mass of C by the atomic weight of carbon: mol_POC = g_POC/12.0107.

**Action:** None taken.

**Reviewer comment: **Fig. 3 Colour differences between samples is hard to decipher.

**Response:** We agree with the reviewer that coloured plots are sometimes hard to interpret quantitatively. However, the purpose of Fig. 3 is to present the general patterns in POC during ATM24 (i.e., higher POC at the start and end of the transect vs. the oligotrophic gyres and higher POC near the surface than at depth). We believe these general patterns are clear in Fig. 3. The dataset was also made publicly available so that the reader can inspect any specific point they may be interested in.

**Action: **None taken.

**Reviewer comment: **P 15, bottom sentence: this statement does not allow for a comparison with the quantitative uncertainty approach conducted by the authors. Please quantify the potential consequence of the large particles.

**Response: **The sentence the reviewer refers to is part of a longer paragraph that might have been broken up in the typesetting process.

**Action: **We have now ensured that the paragraph is all connected.
